# Supplementary figures and images for: Genes in human obesity loci are causal obesity genes in C. elegans
Source: PLoS Genet. 2021 Sep 7;17(9):e1009736. doi: 10.1371/journal.pgen.1009736 (PMC8462697; doi:10.1371/journal.pgen.1009736)

S1 Figure

A

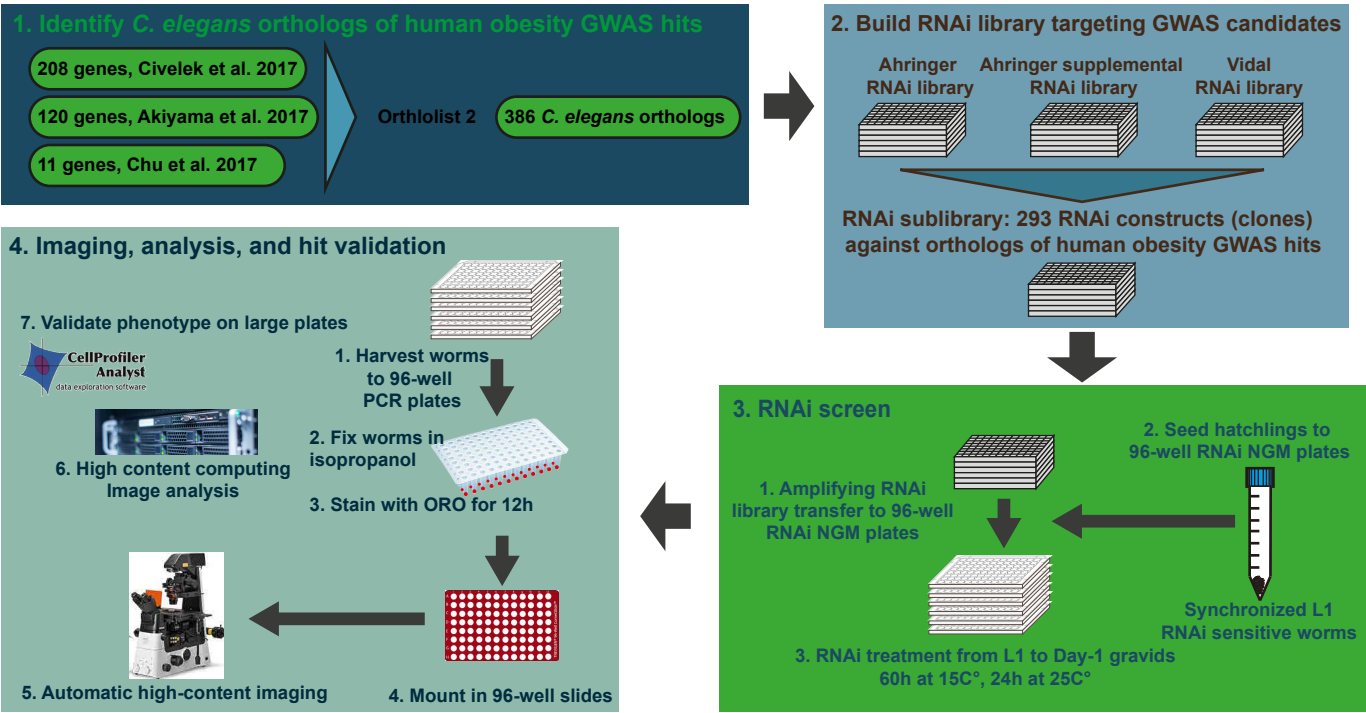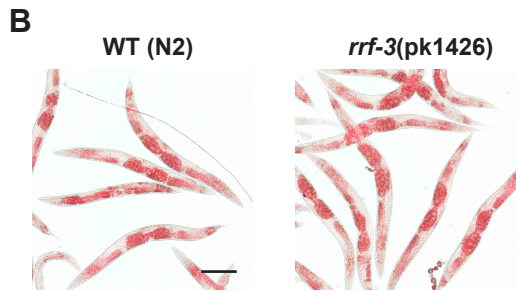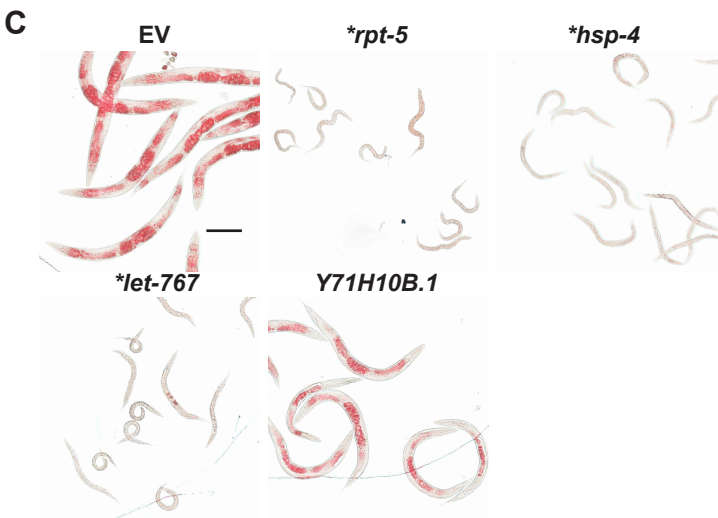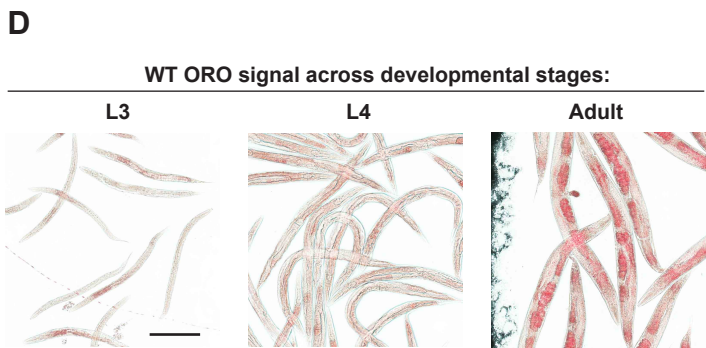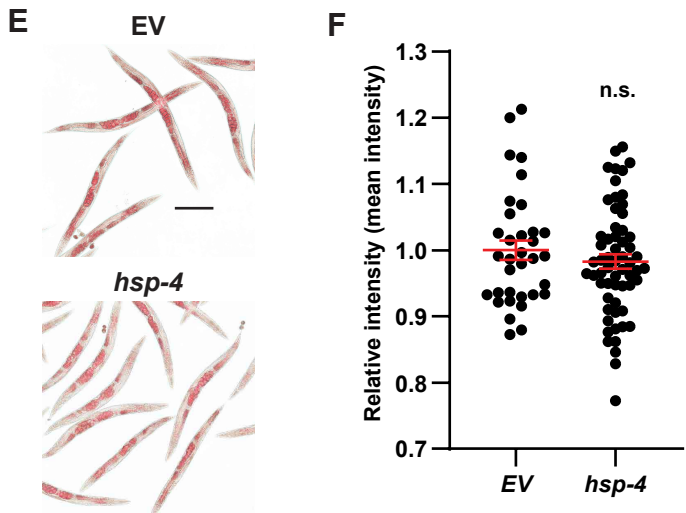

Supplement: S1 Fig — Throughout this figure: Scale bars = 200μm, Error bars = S.E.M. N = number of independent biological replicates. Statistical significance was assessed via ratio t-test, *p≤ 0.05, **p≤0.01, ***p≤0.001 and ****p≤0.0001. (A) Overview of the workflow of the screen for genes altering fat storage in C. elegans. (B) Representative images of the body fat content and distribution in WT(N2) and rrf-3 RNAi sensitive mutant worms. N = >5. (C) Representative images of the body fat content and distribution as made evident with ORO in worms treated with RNAi against the obesity candidate genes from the L1 stage. Asterisks denote those RNAi treatments that led to developmental delay. (D) Representative images of the age-dependent increases in fat stores as revealed by staining L3, L4, and 1-day adult worms with ORO. N = >5. (E) Retesting of ORO staining in worms treated with hsp-4 RNAi from the L3 stage showed no effect of hsp-4 knockdown on C. elegans body fat content. (F) ORO quantification in worms treated with hsp-4 RNAi as represented in panel E. Each data point represents the ORO intensity in one worm. Individual intensity values were normalized to the mean value of the EV RNAi control. N = 3. (PDF) [file pgen.1009736.s001.pdf]

S2 Fig

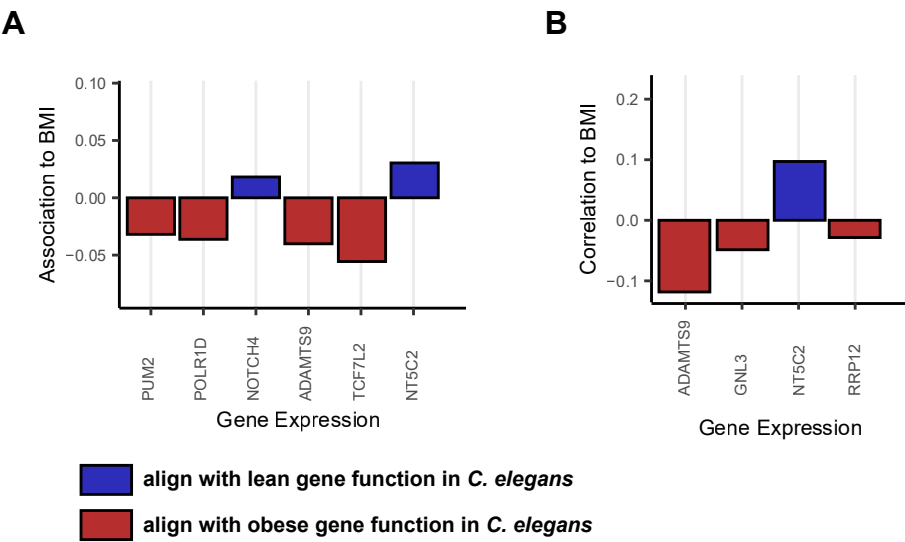

Supplement: S2 Fig — A) Association to BMI in TWINsUK B) Correlation with BMI in GTEx. Blue indicates the direction of association aligns with the lean gene in C. elegans. Red indicates the direction of association aligns with the obese gene in C. elegans. (PDF) [file pgen.1009736.s002.pdf]

S3 Fig

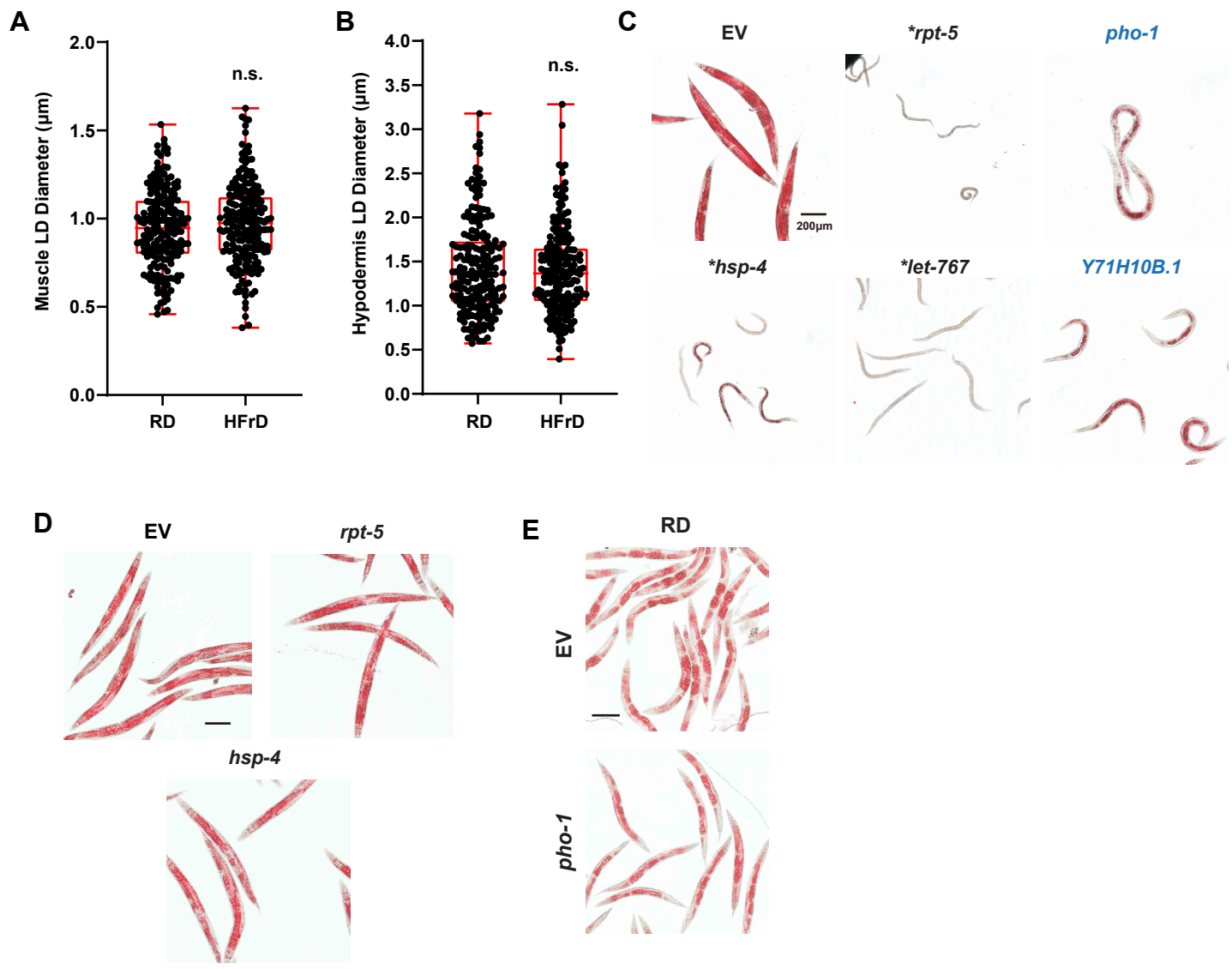

Supplement: S3 Fig — Throughout this figure: Error bars = S.E.M. N = numbers of independent biological replicates. Unless specified, unpaired nonparametric t-tests were used to assess the significance. *p≤ 0.05, **p≤0.01, ***p≤0.001 and ****p≤0.0001. (A) Quantification of muscle LD size in worms fed RD or HFrD. Each data point represents the measurement of random LDs in the images. ≥10 worms were measured in each independent biological replicate. N = 3. (B) Quantification of hypodermis LD size in worms fed RD or HFrD. Each data point represents the measurement of a random LD in the images. ≥10 worms were measured in each independent biological replicate. N = 3. (C) Body fat content of worms treated with RNAi against all of the DIO suppressors observed in the primary screen. RNAi was initiated from the L1 stage. pho-1 and Y71H10B.1 (Blue font) are the 2 DIO suppressors that cause leanness without developmental delay. (D) Representative images of the body fat content and distribution in worms fed HFrD from the L1 stage but treated with RNAi against rpt-5 and hsp-4 from the L4 stage. No change in fat content was observed in this condition. N = 3. (E) Representative images of the body fat content and distribution in worms treated with RNAi against pho-1 from the L4 stage. No change in fat content was observed. N = 3 (PDF) [file pgen.1009736.s003.pdf]

S4 Fig

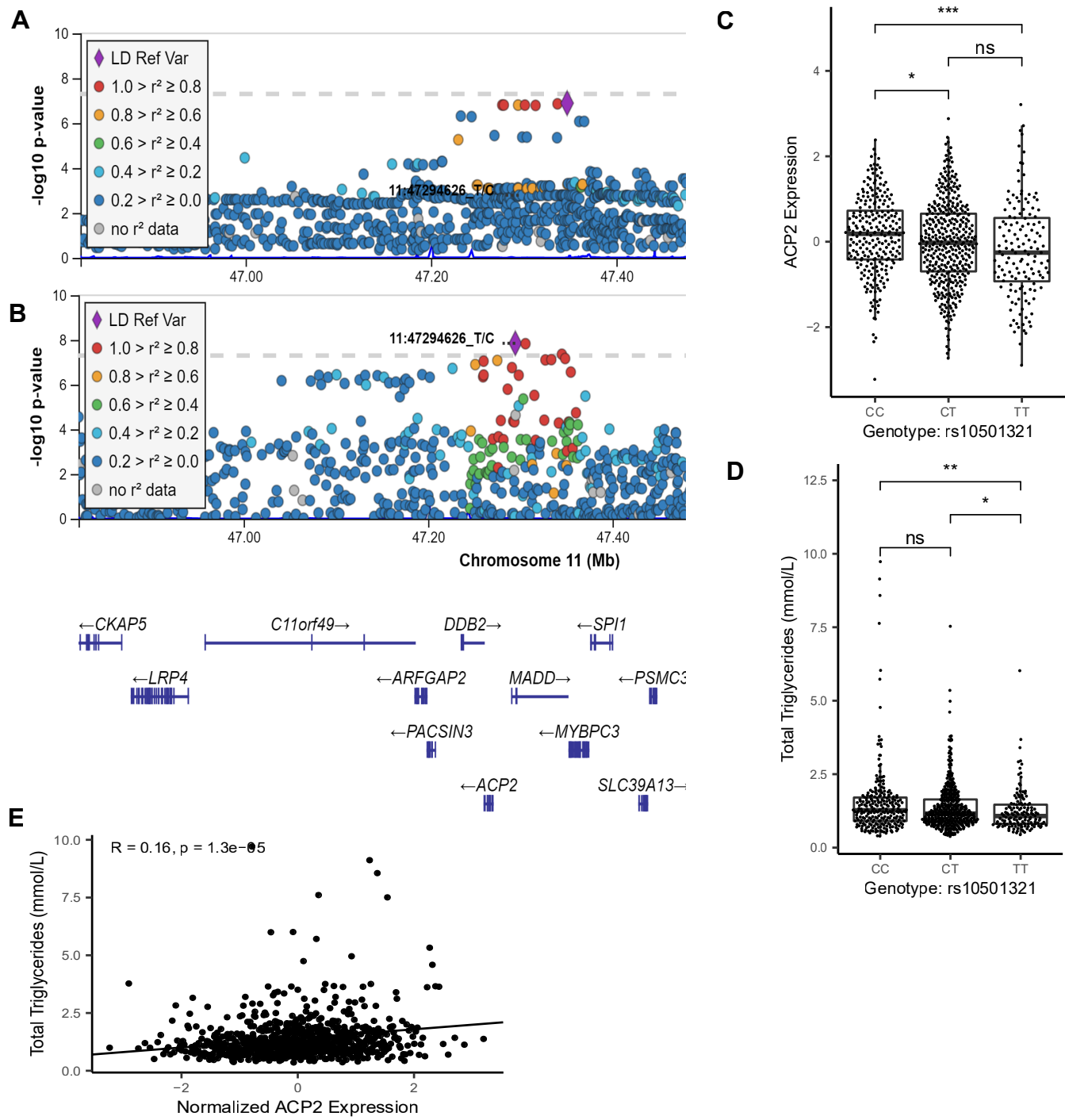

Supplement: S4 Fig — LocusZoom plots of the associations of the SNPs near ACP2 with (A) BMI and (B) ACP2 gene expression in subcutaneous adipose tissue in the METSIM cohort. The effect of the alleles of rs10501321 on (C) ACP2 expression and (D) total triglycerides. (E) Correlation between BMI and ACP2 (human pho-1 ortholog) expression in the METSIM cohort. (PDF) [file pgen.1009736.s004.pdf]

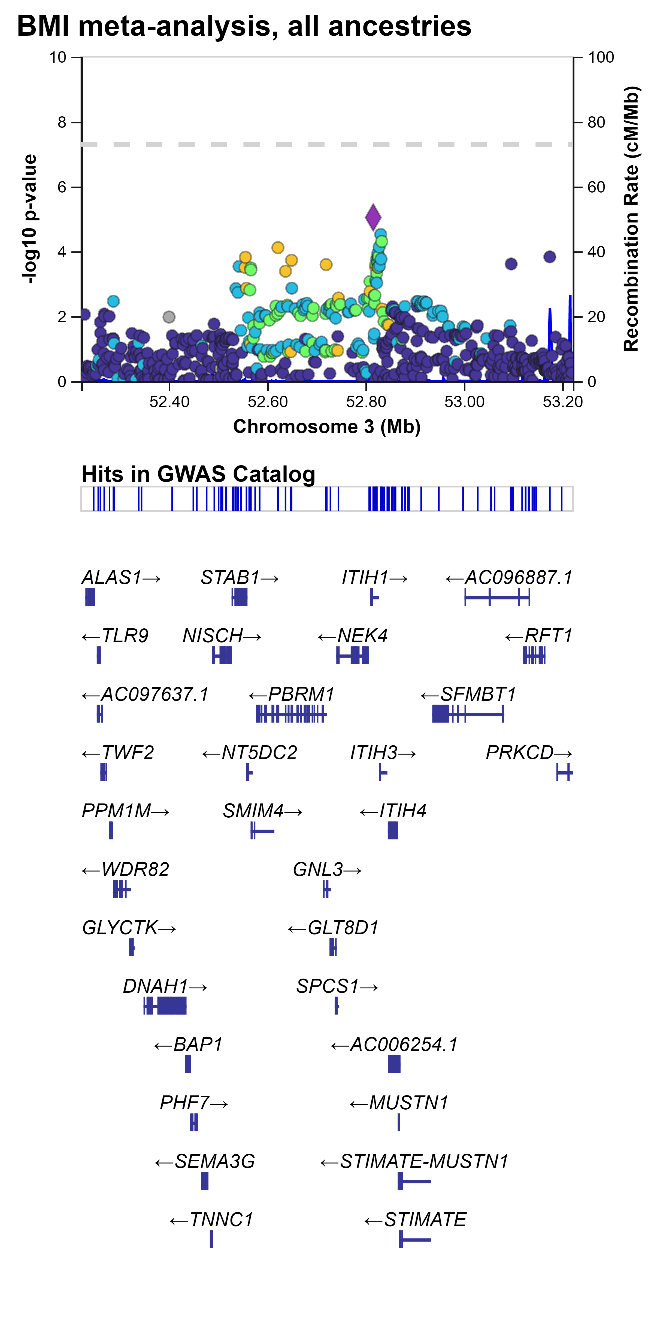

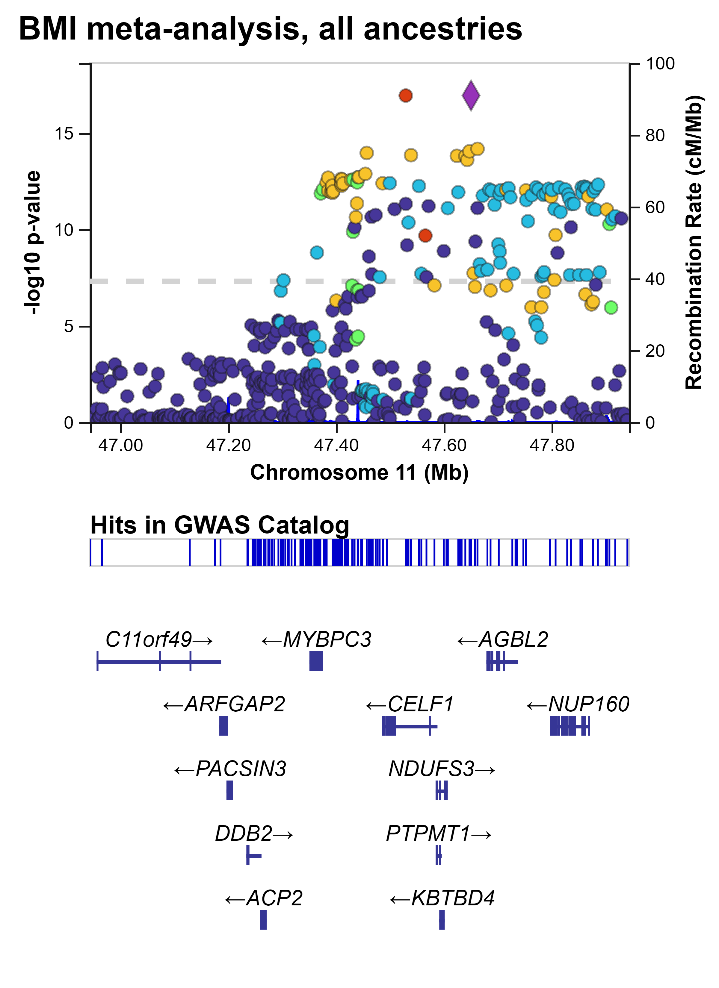

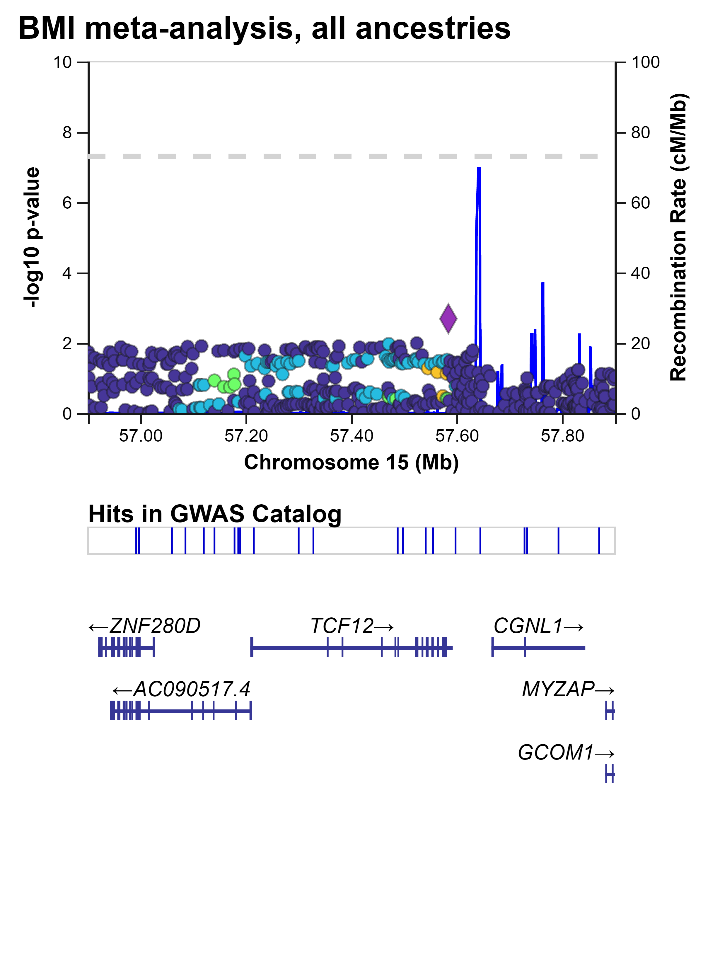

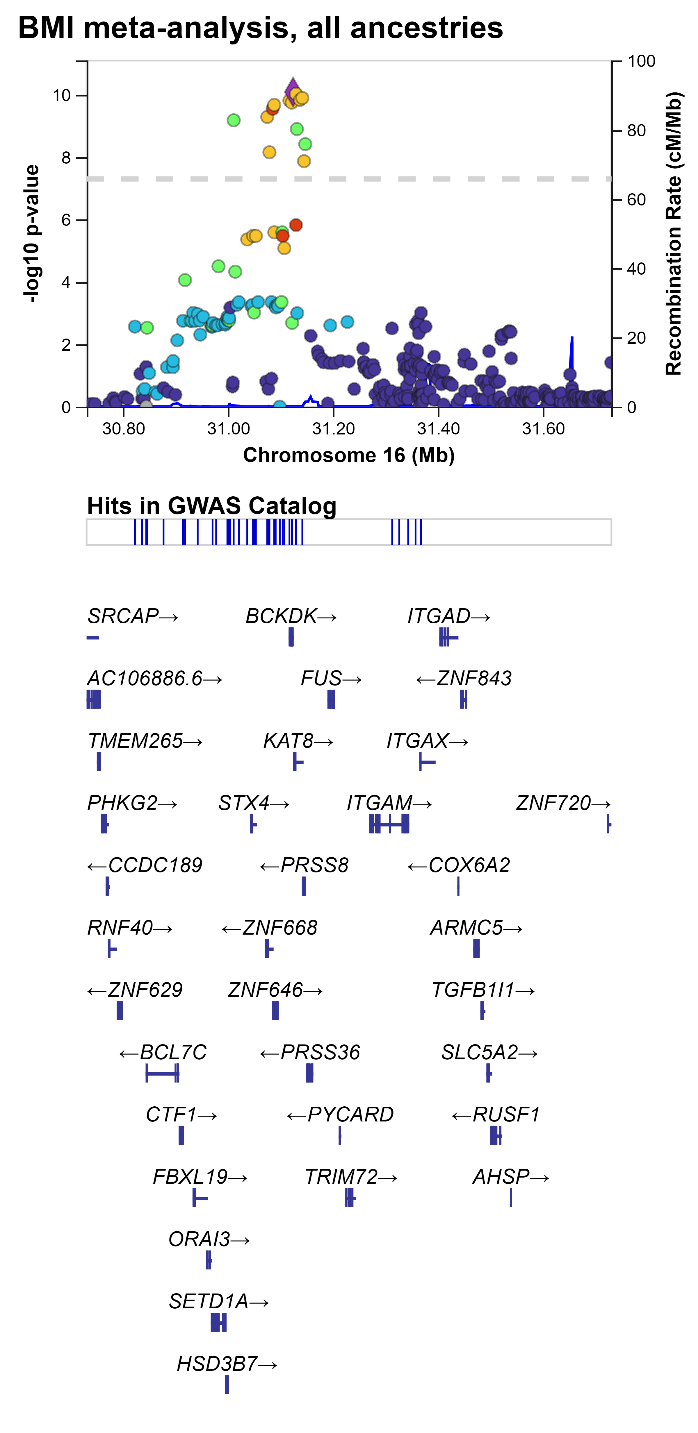

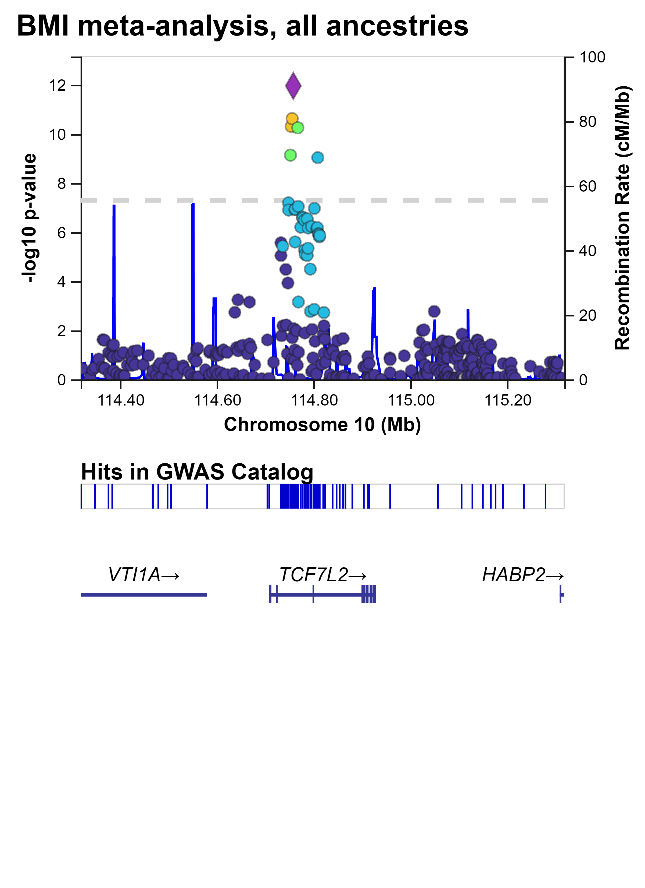

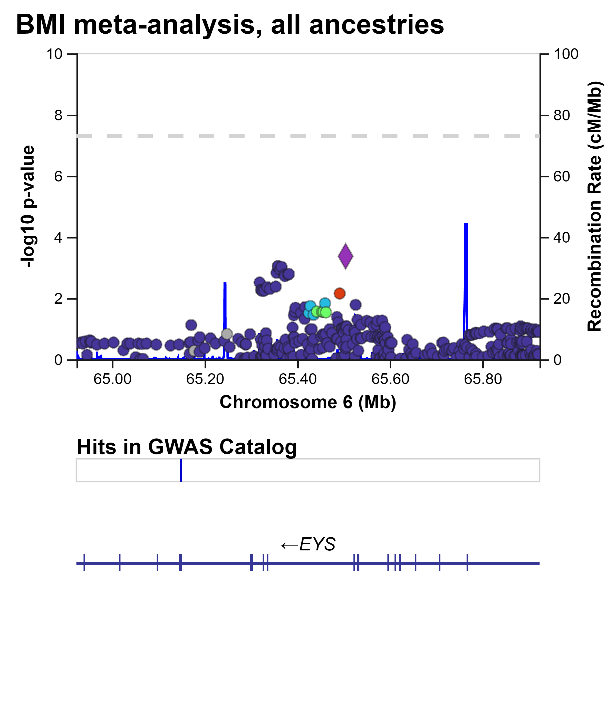

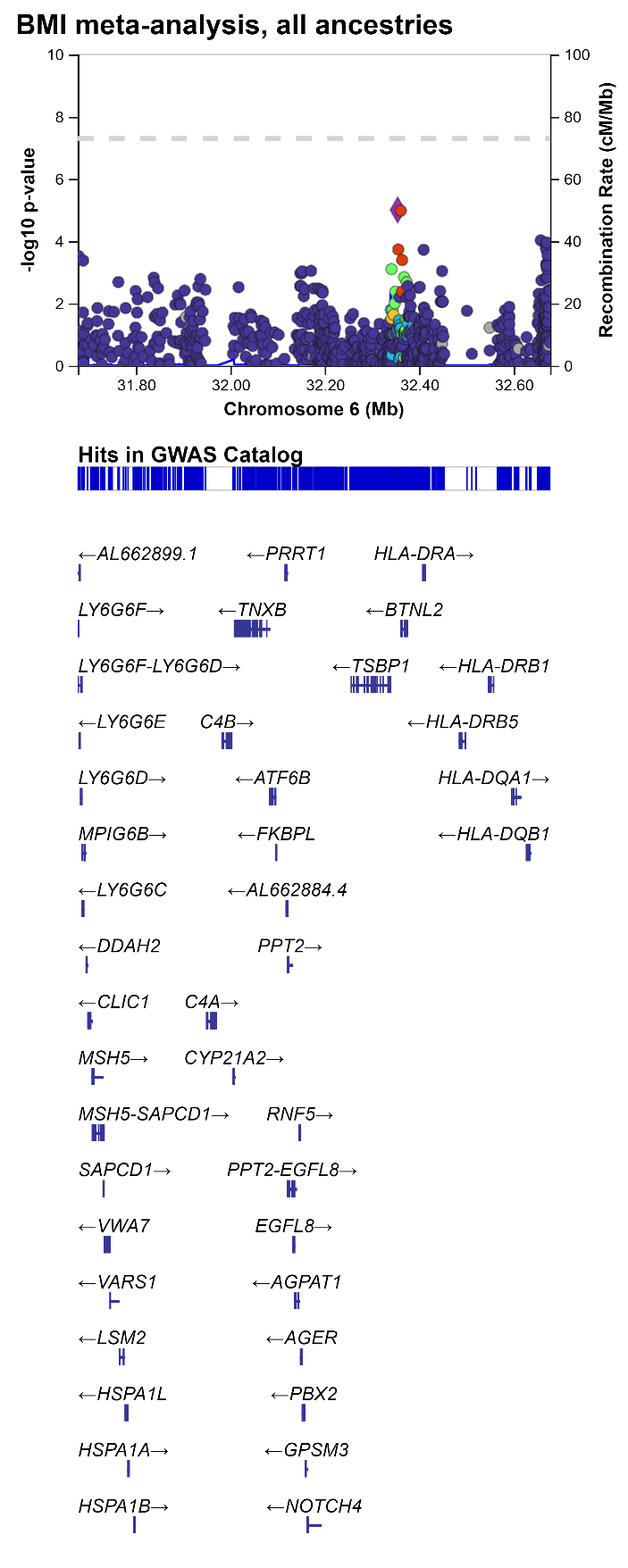

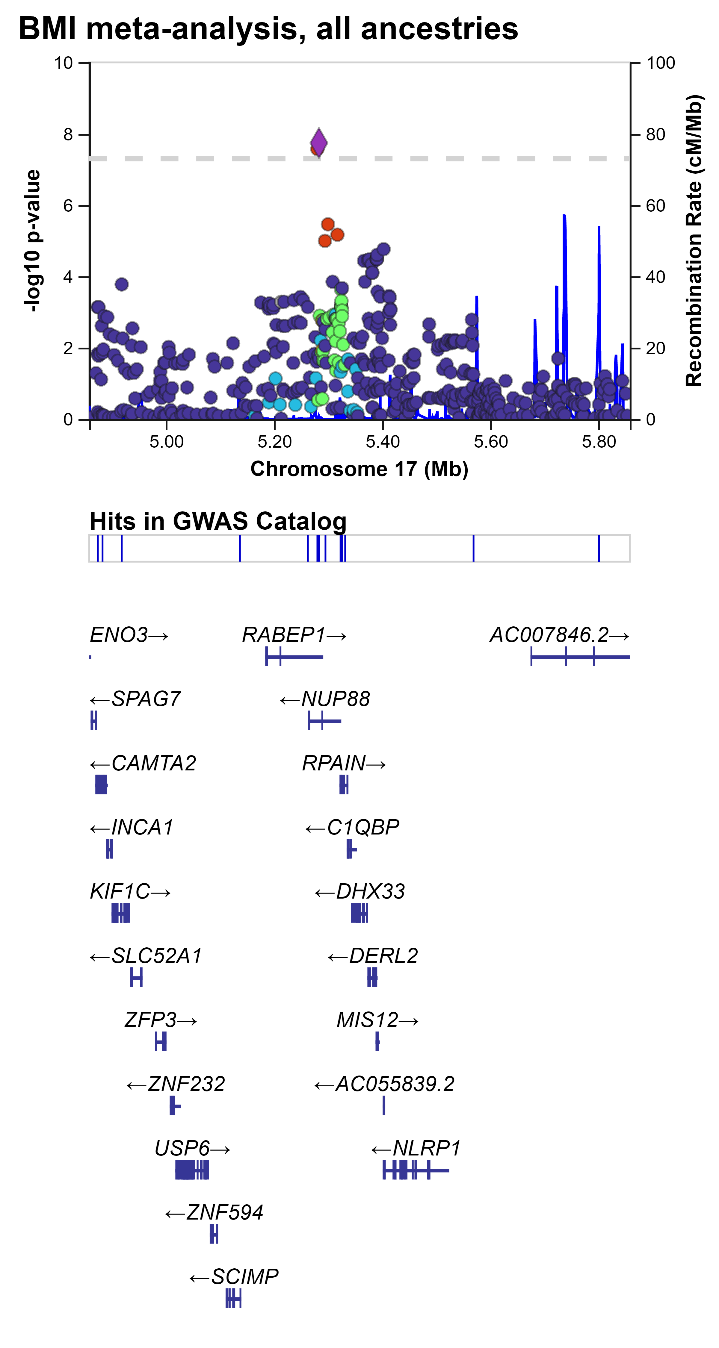

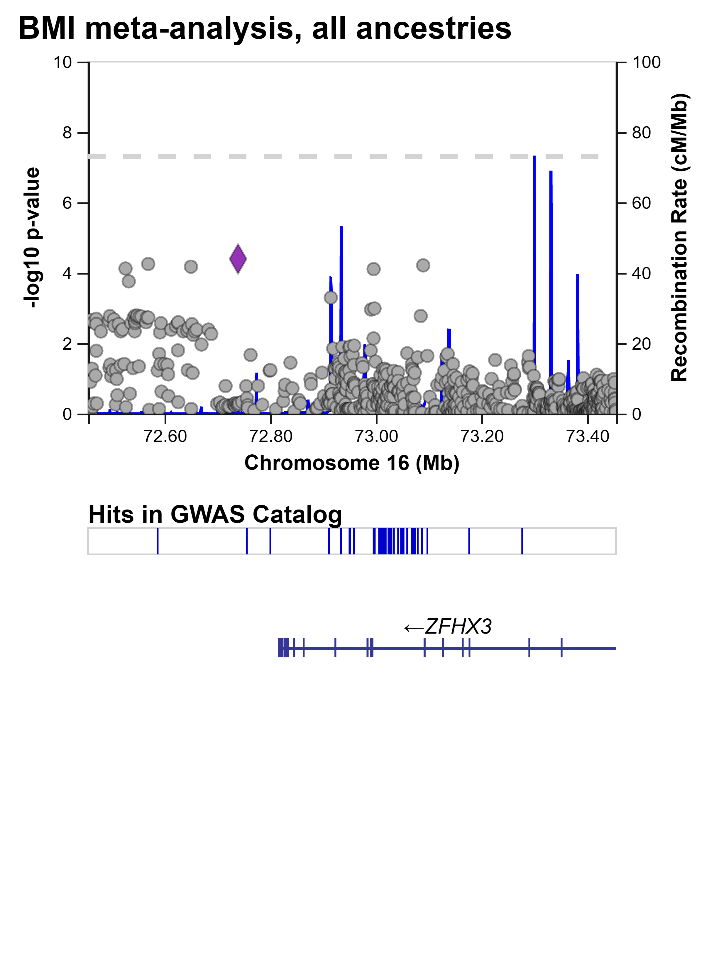

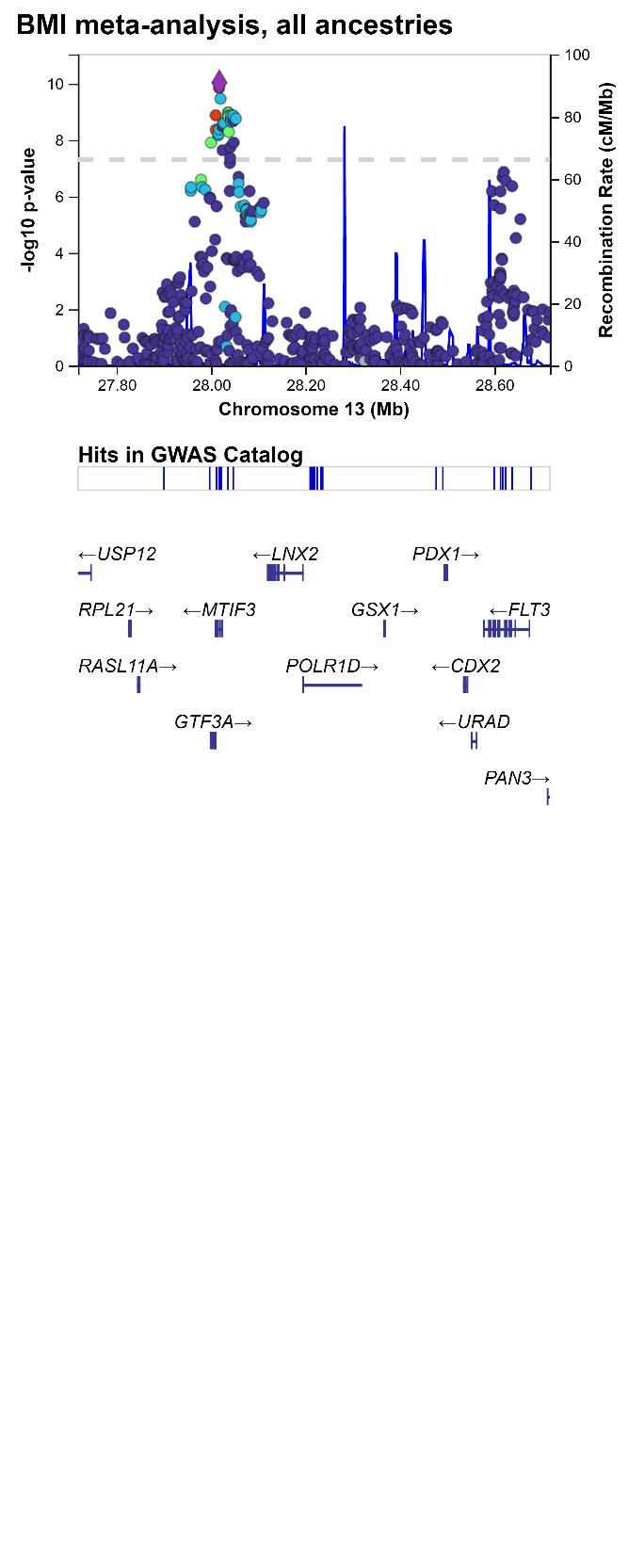

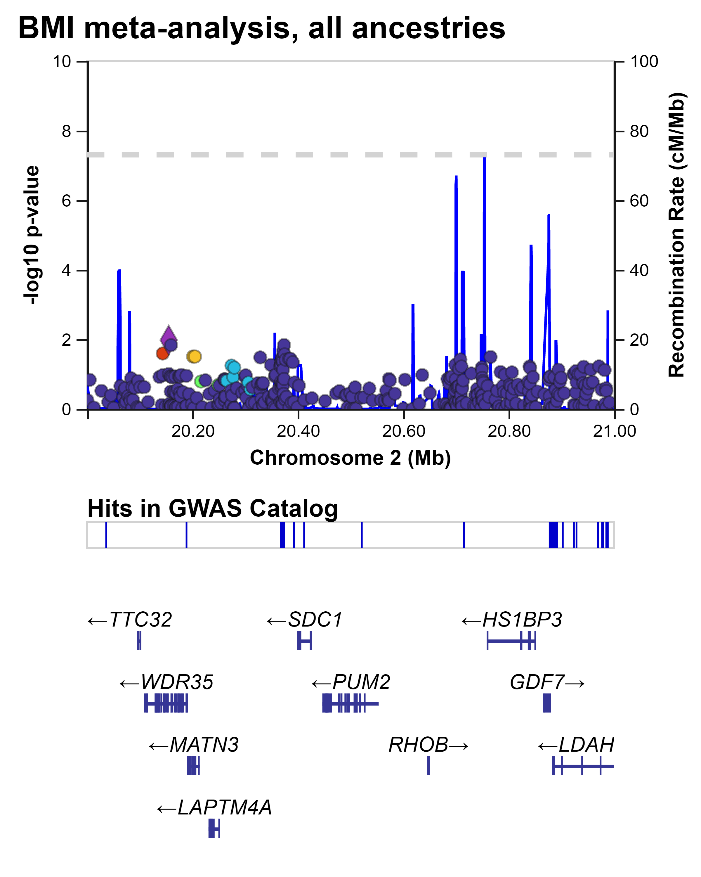

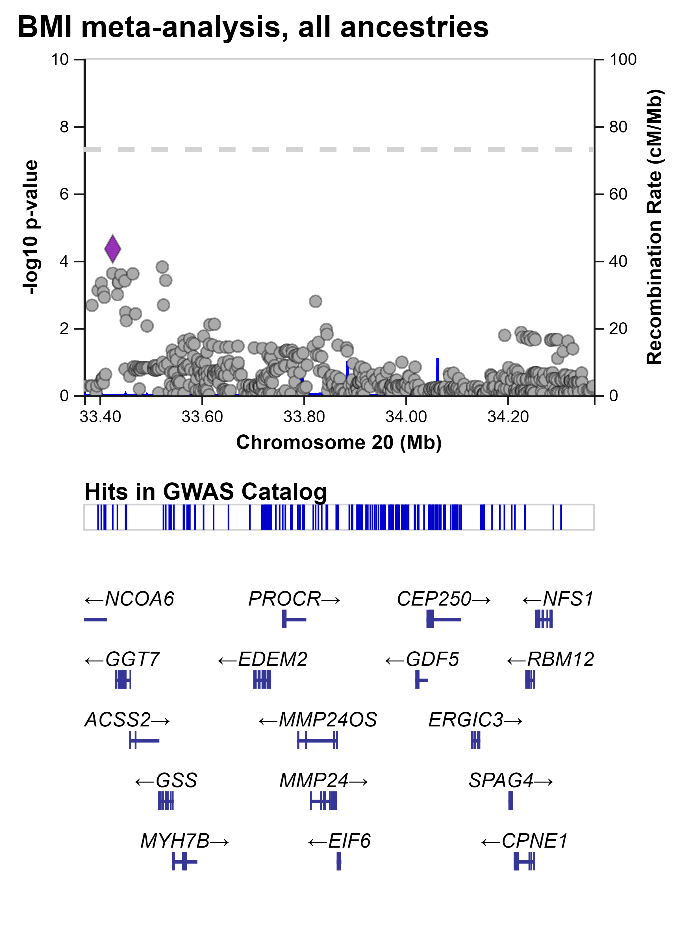

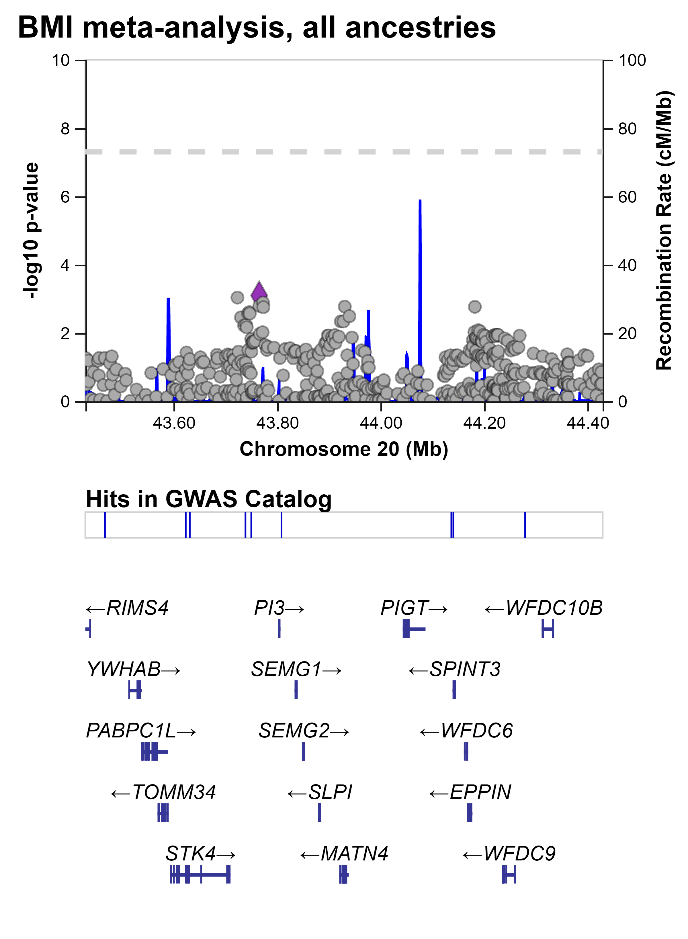

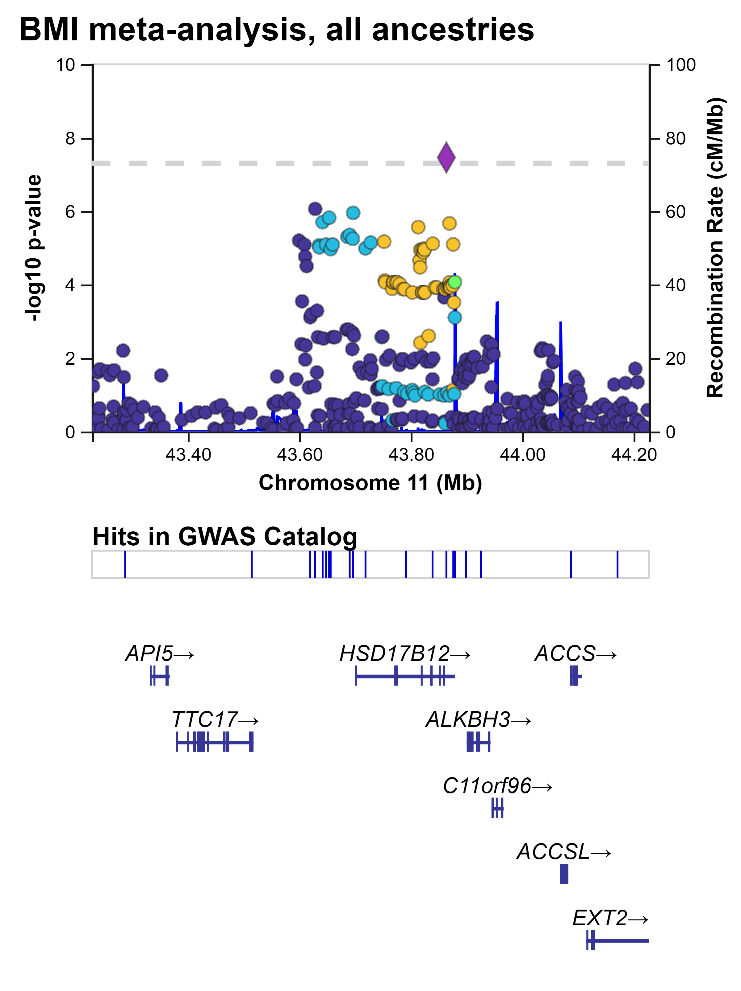

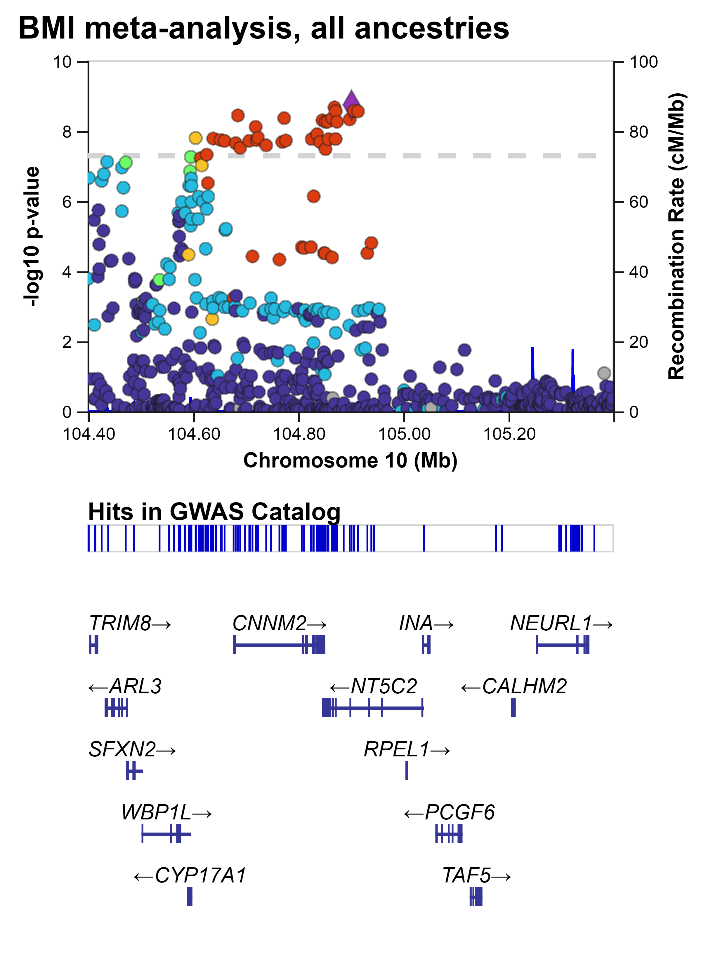

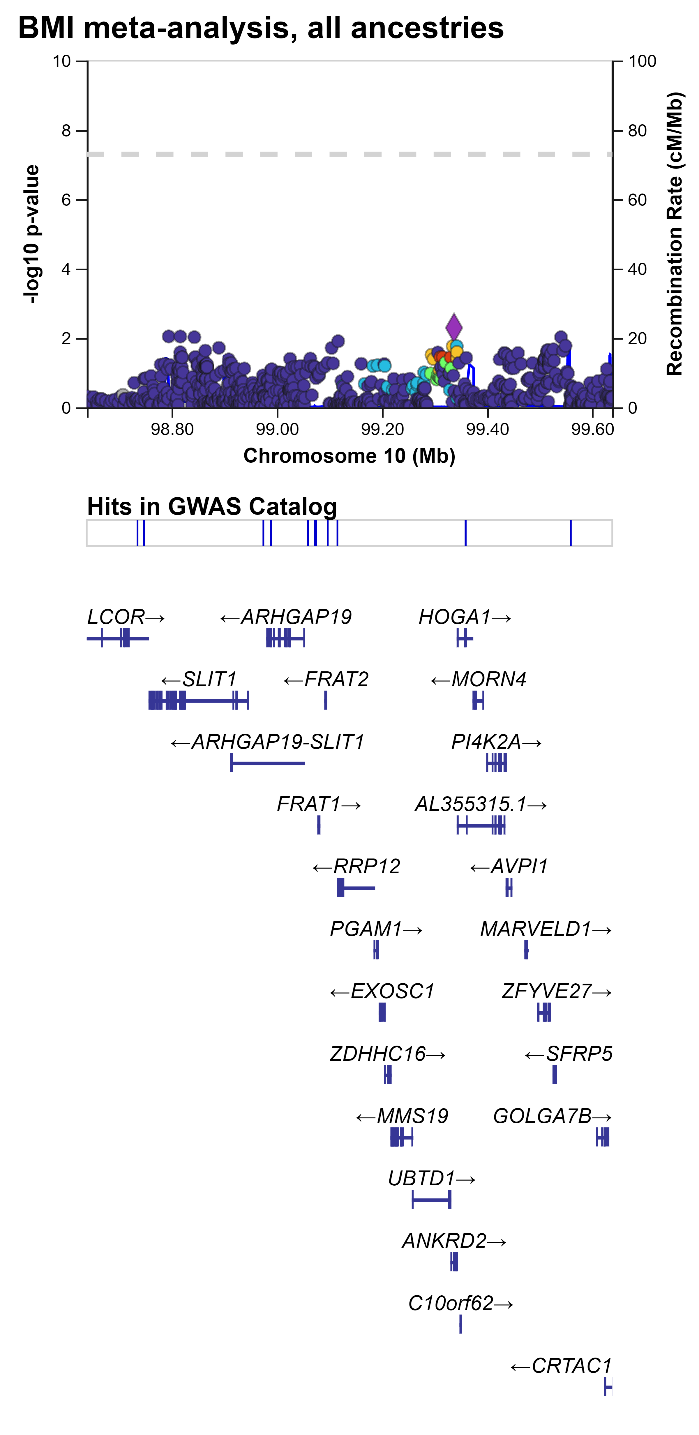

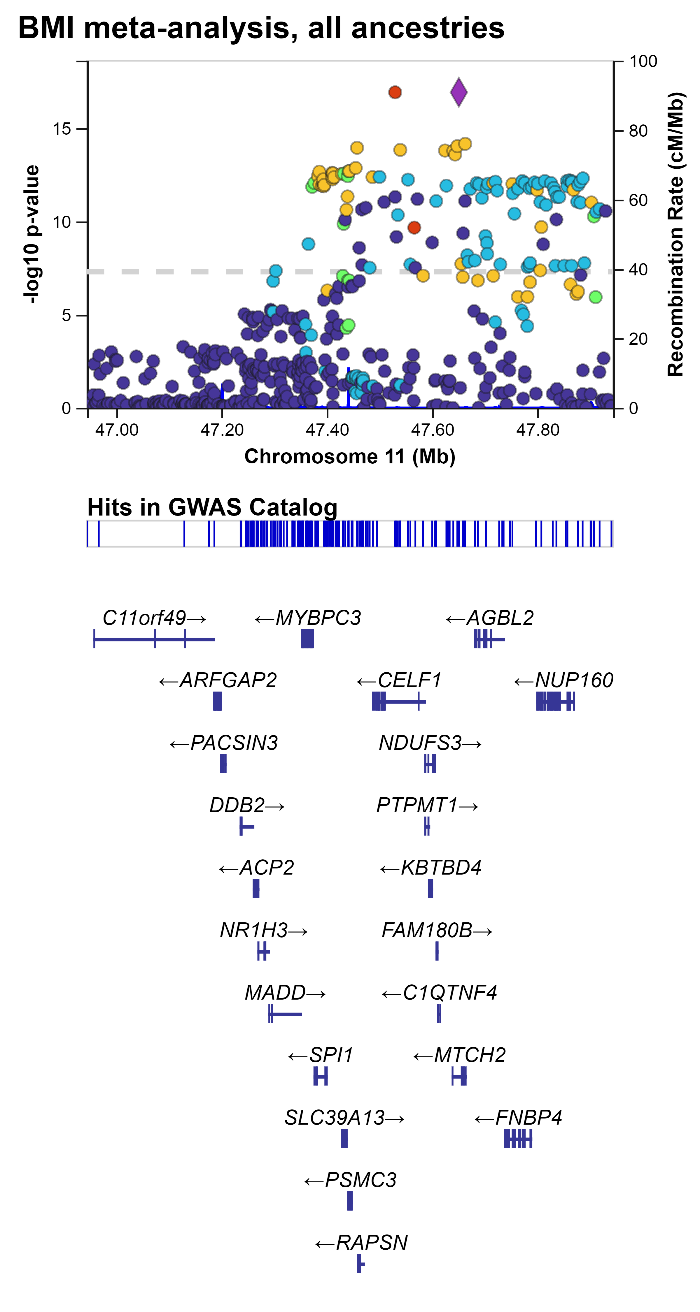

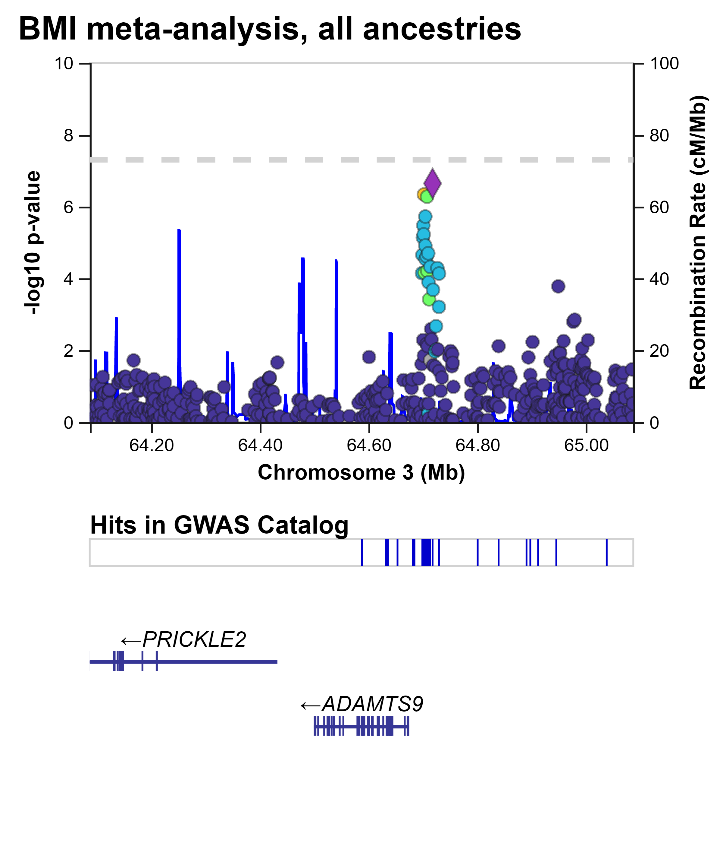

Supplement: S1 LocusPlots — (DOCX) [file pgen.1009736.s008.docx]

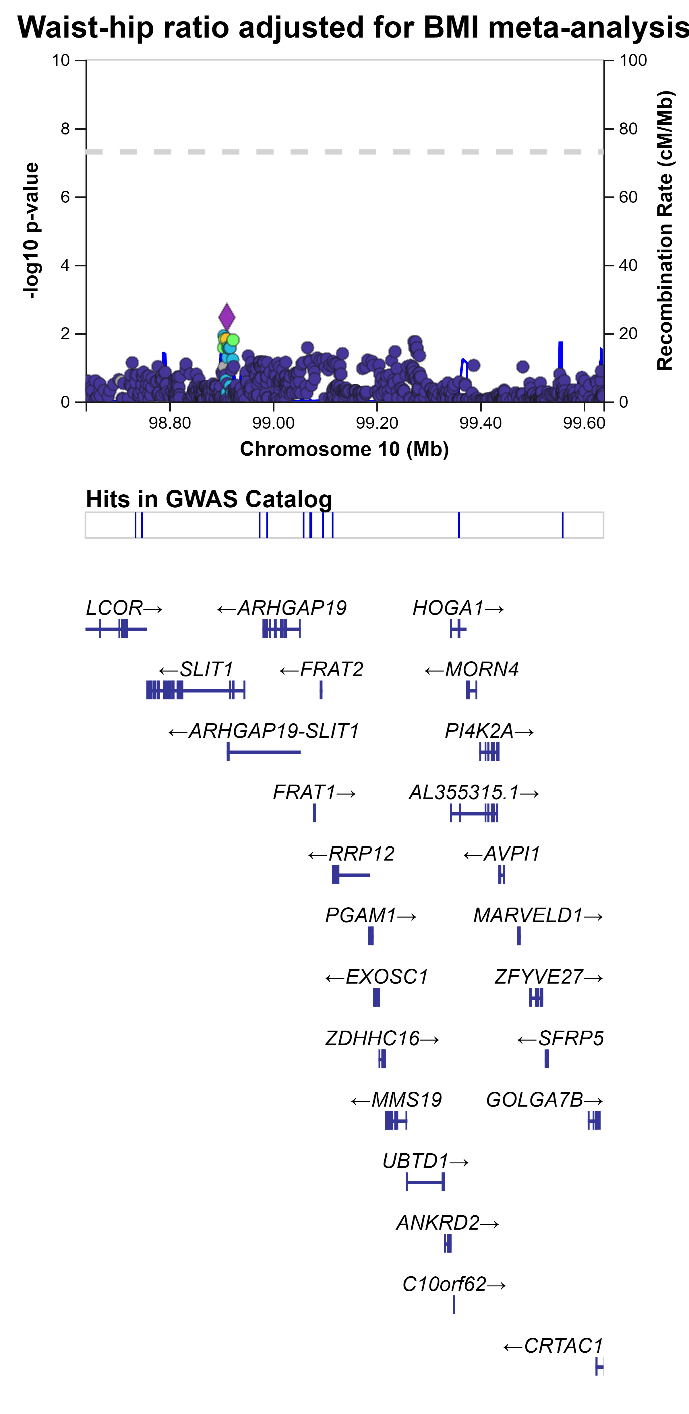

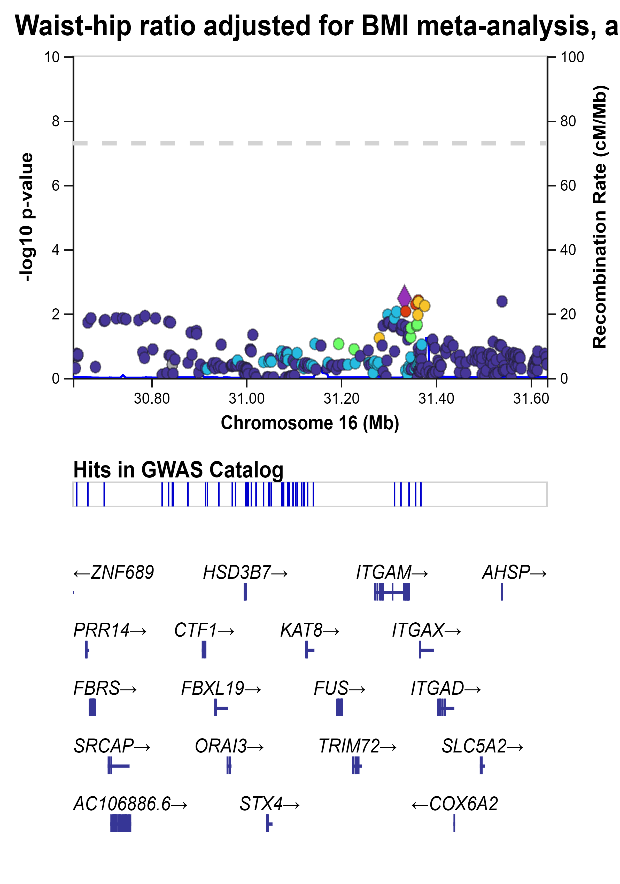

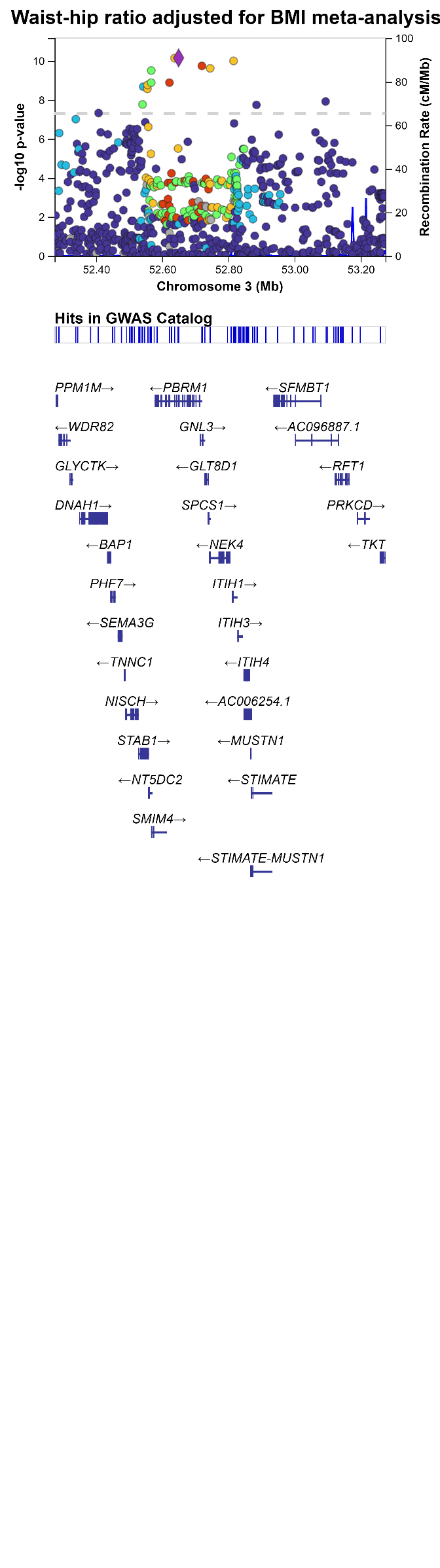

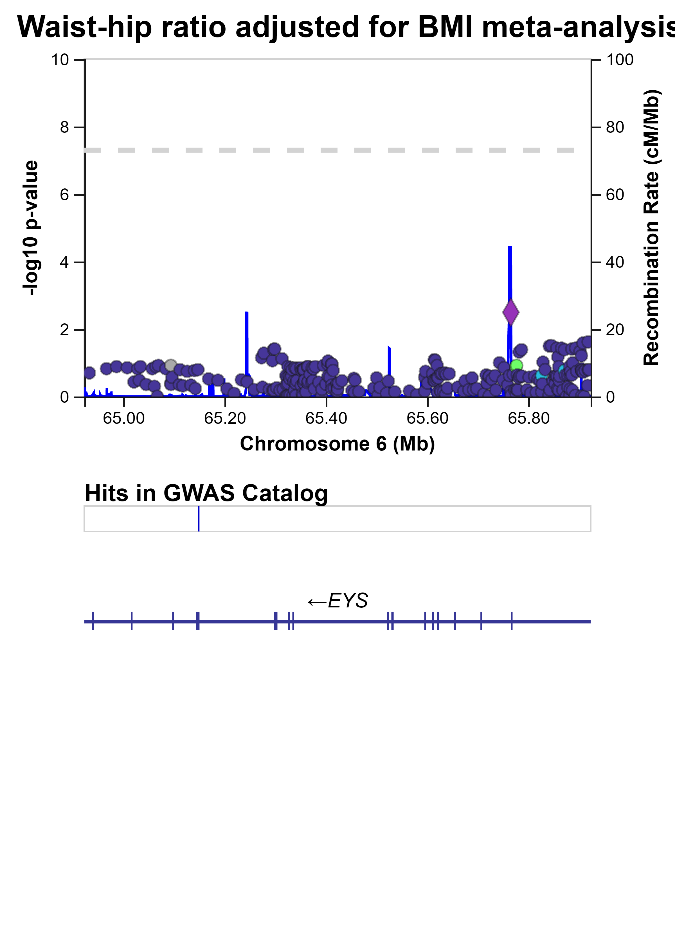

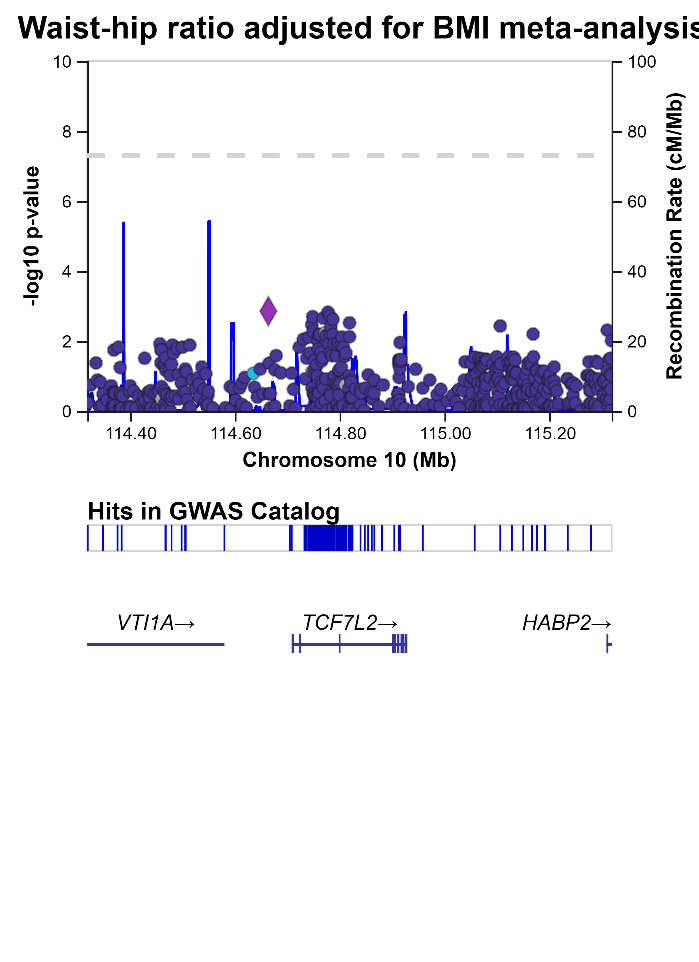

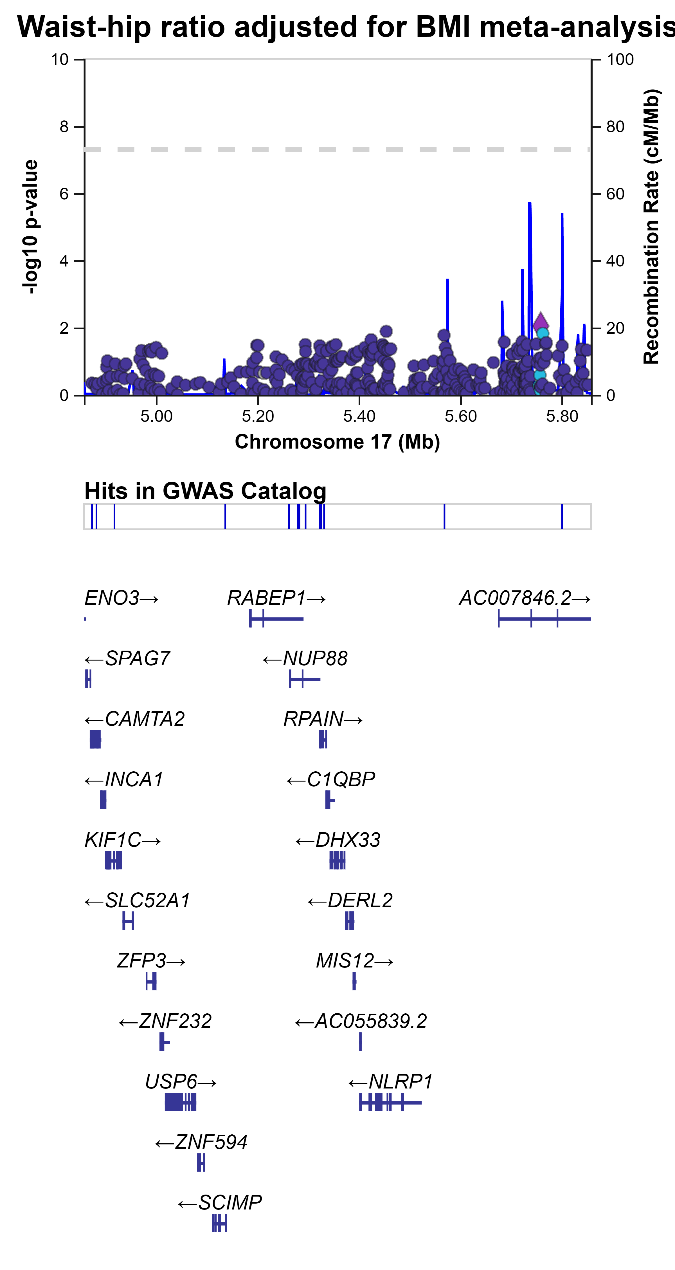

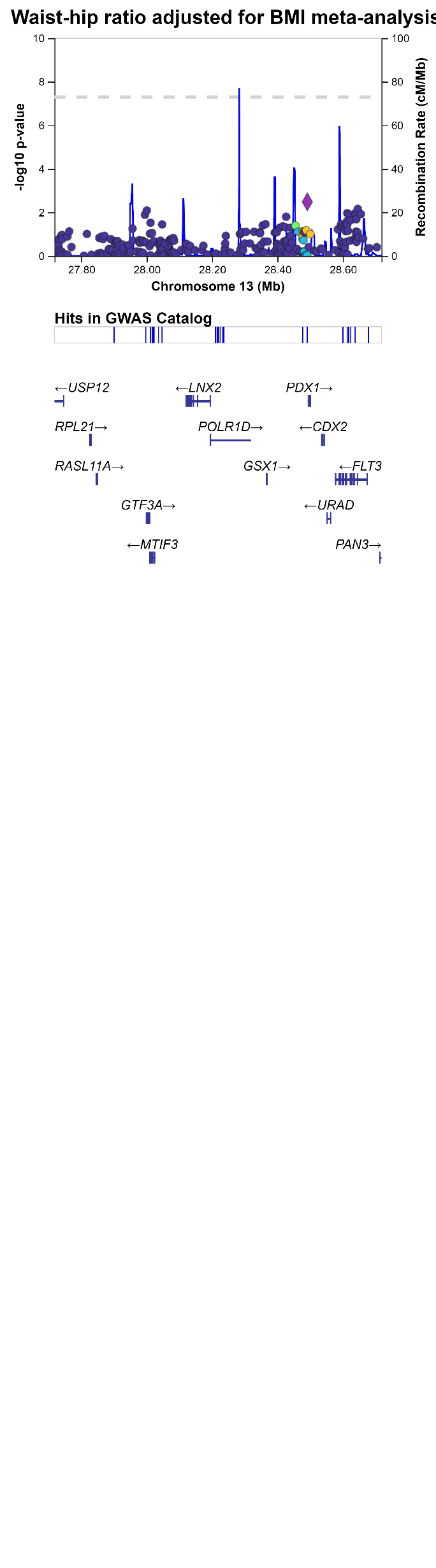

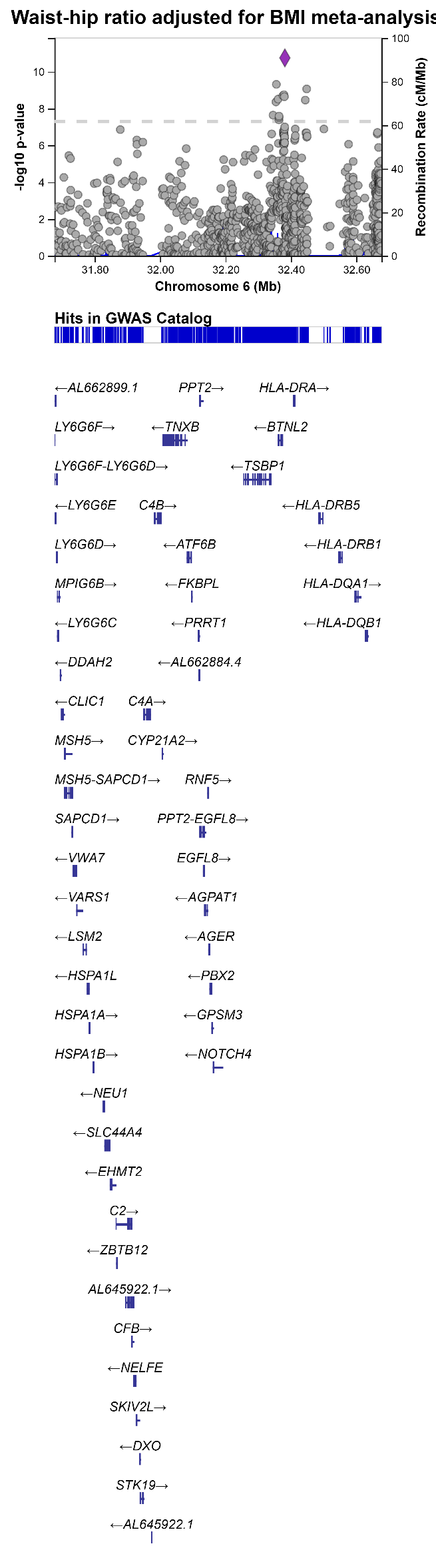

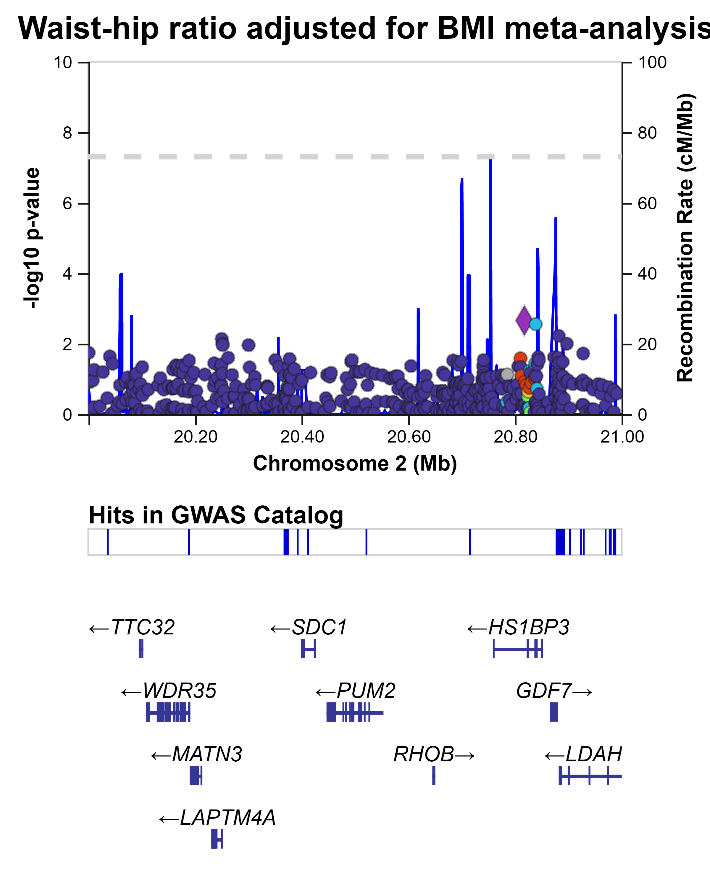

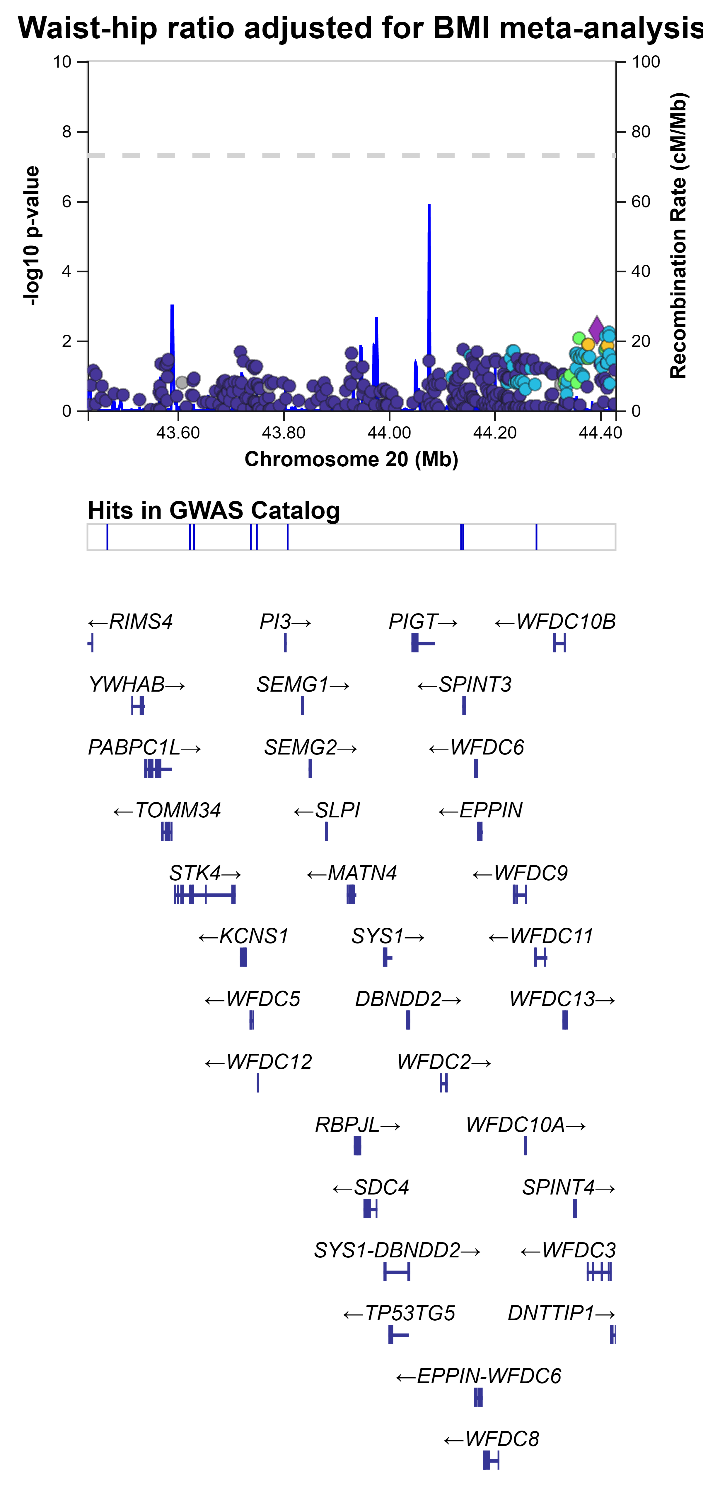

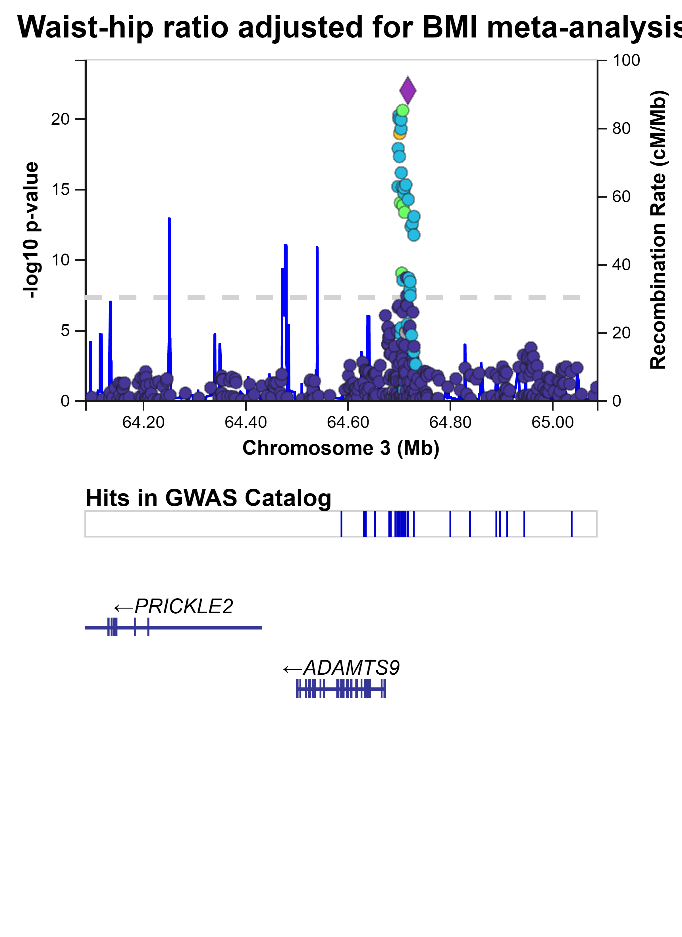

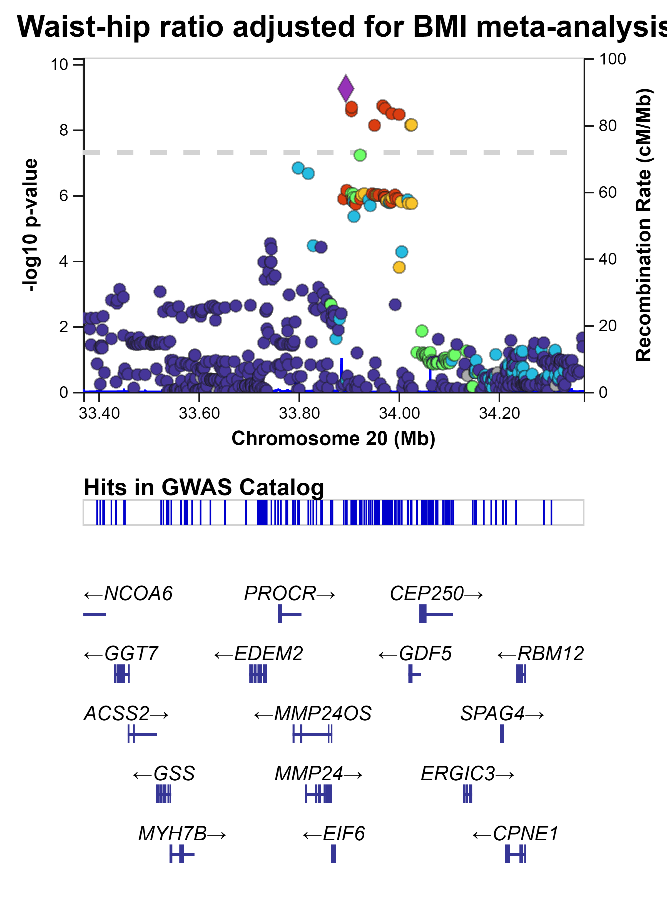

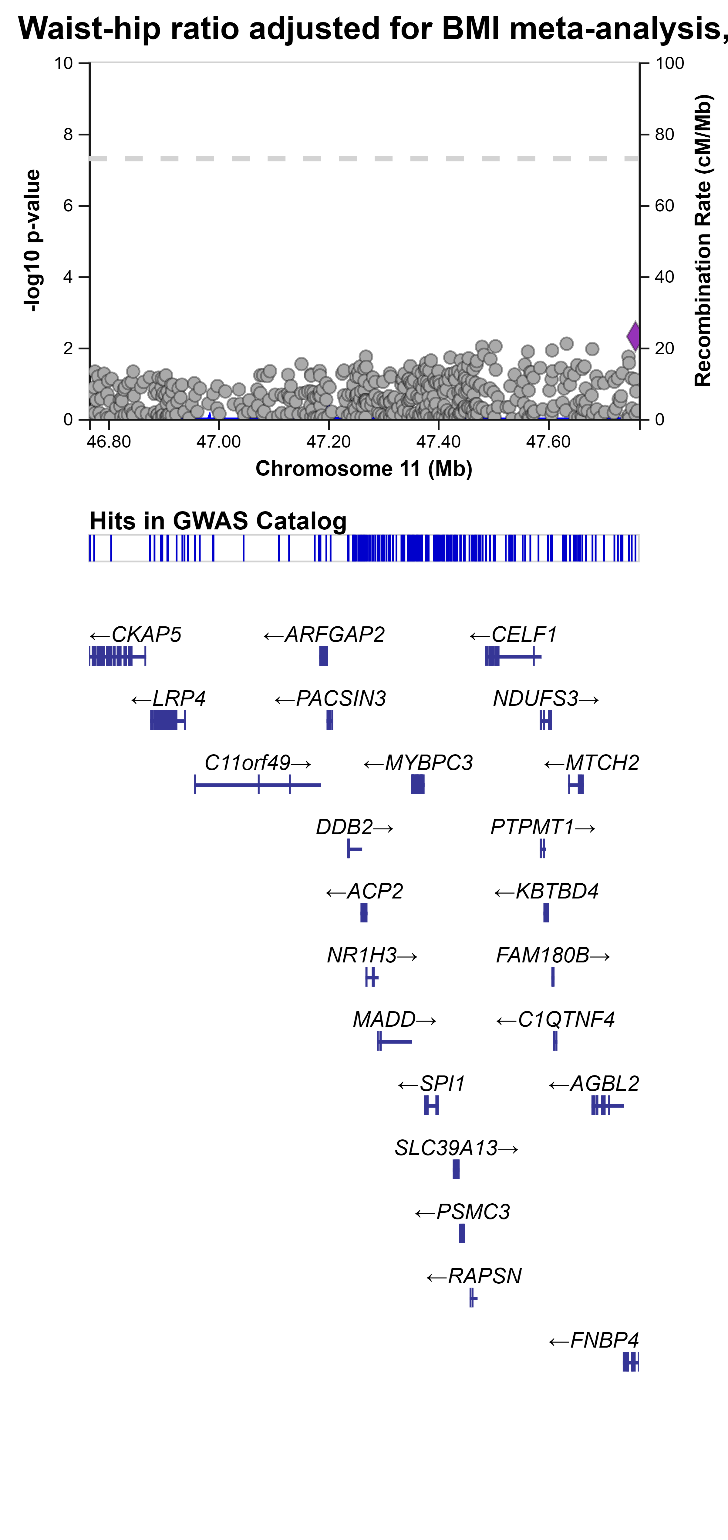

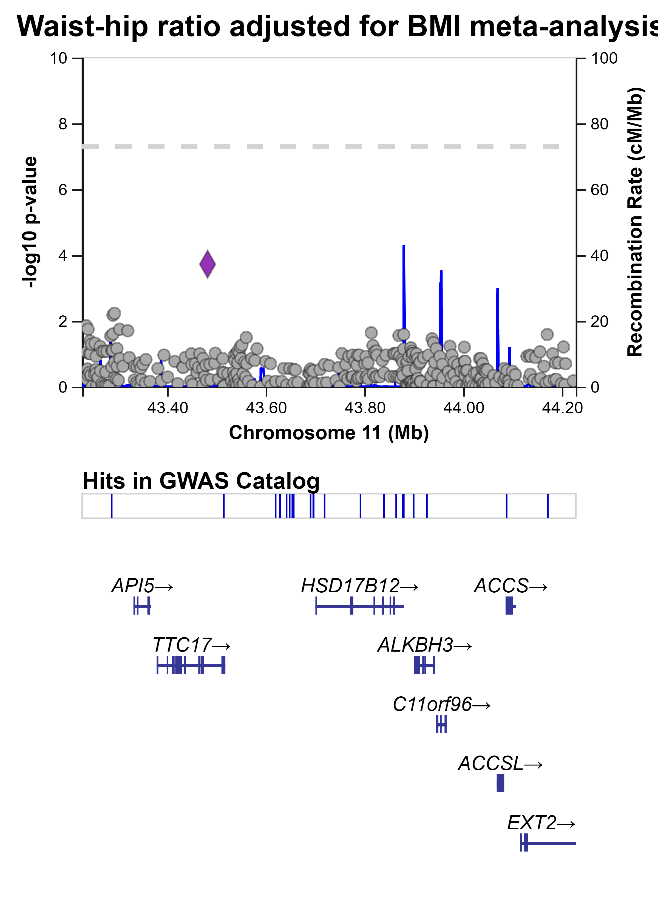

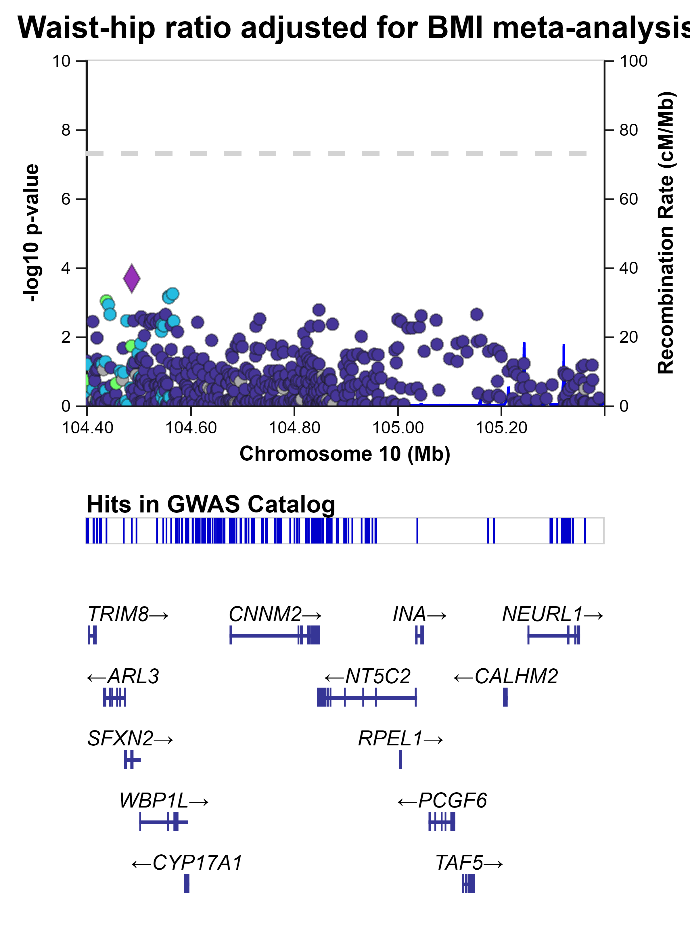

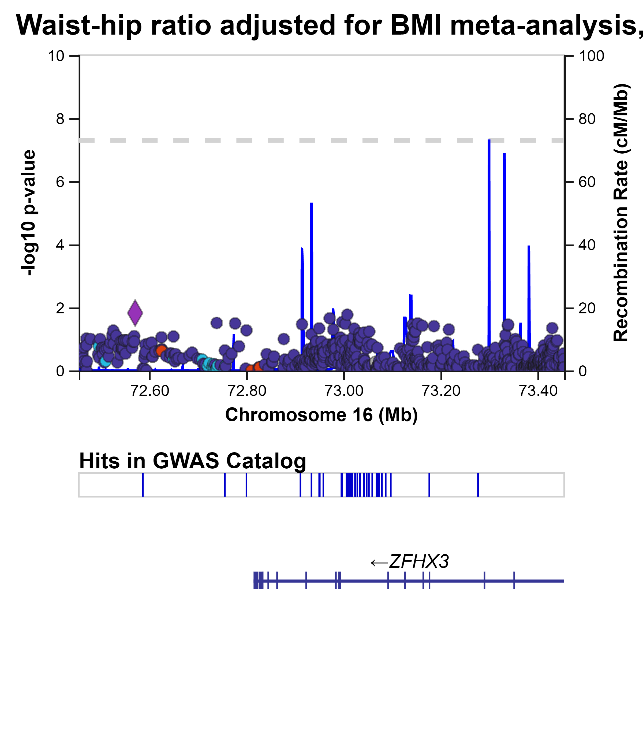


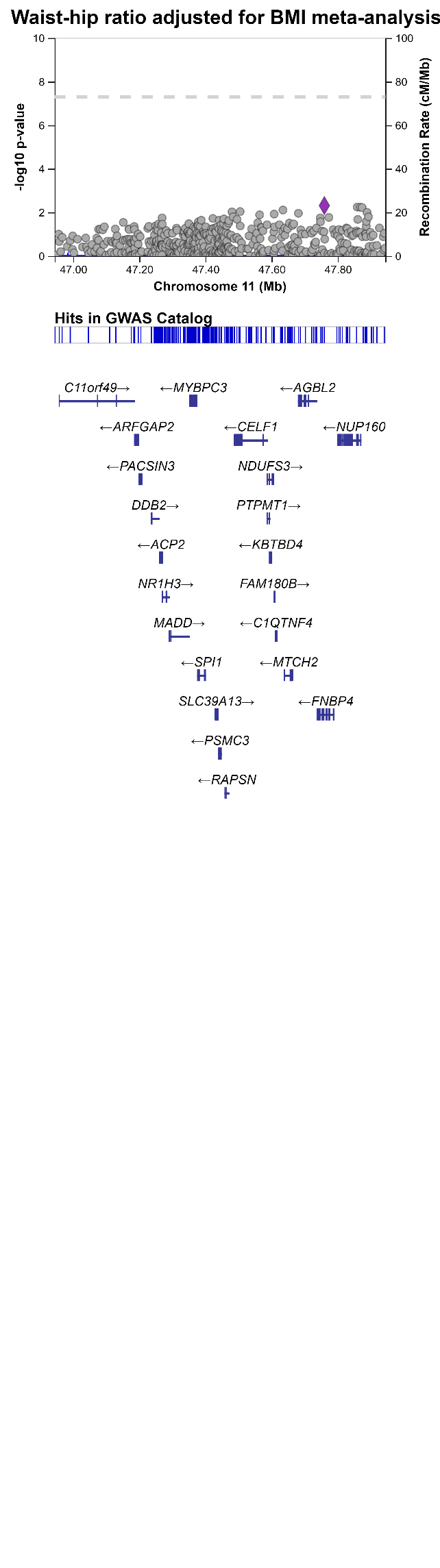

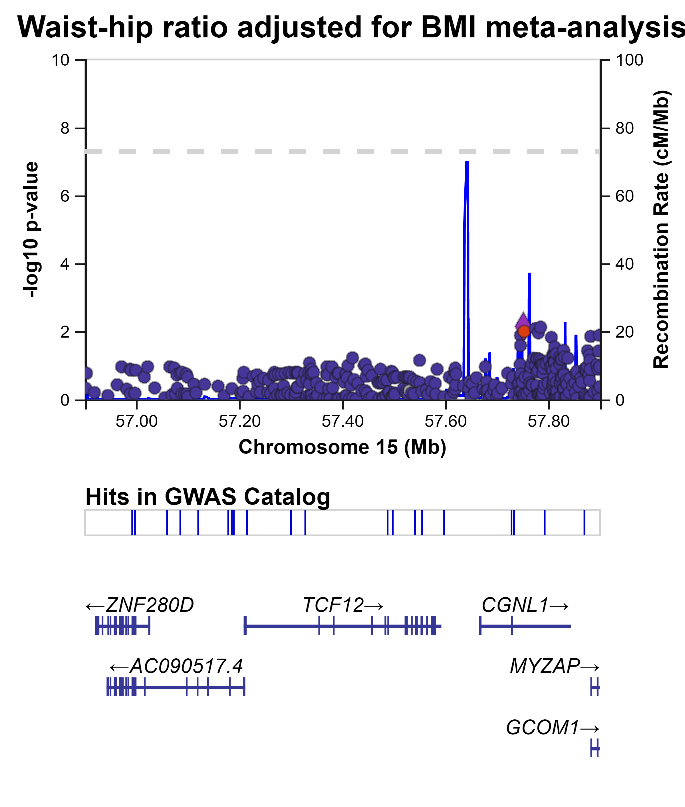

Supplement: S2 LocusPlots — (DOCX) [file pgen.1009736.s009.docx]
