# Supplementary material for: Genes in human obesity loci are causal obesity genes in C. elegans
Source: PLoS Genet. 2021 Sep 7;17(9):e1009736. doi: 10.1371/journal.pgen.1009736 (PMC8462697; doi:10.1371/journal.pgen.1009736)
Supplement: Attachment — Submitted filename: Ke_PlosGenetics_Rebuttal_V3.pdf [file pgen.1009736.s012.pdf]

Point by point response to reviewer 1

(All changes to address reviewer 1's comments are highlighted in green in the new version of the manuscript)

Minor criticisms:
1. RNAi of the gene rpt-5 caused different fat defects depending on when the gene was inhibited during C. elegans development. The authors decided to classify this gene with 13 post-developmentally obese genes. However, the authors did not specify whether the plate-based RNAi, where 13 of 14 genes retested the 96-well assay phenotype, was performed on L4 animals. It did not seem that way in the text. So, the authors should specify that the RNAi for these 14 genes (not rpt-5) was post-L4 or be more specific in their classification.
We thank the reviewer for pointing to this mistake.<br><br>We erroneously labelled all obesity genes as post-developmentally obese even though the only gene of the group that was tested using post-developmental RNAi was *rpt-5*. This was the only obese gene tested post-developmentally because it was the only one showing developmental delay in the primary (96-well) RNAi screen in which RNAi treatment was started from the L1 stage.<br><br>Please find the revised text in lines 216-217 and 250-254.
2. High-fructose diet induces behavior effects similar to A-beta over expression. However, that similarity does not mean that they act in the same pathway. The result is a mere correlation. The authors should tone down their conclusions from lines 394-404 and in parts of the Discussion.
We completely agree with the reviewer. Toned down conclusions can now be found in lines 452-454.
Specific edits:
Line 98, space between association
Change was made (lines 97-98).
From Line 103 and throughout, e.g. should be italicized and have a comma
Format was changed throughout the text (highlighted in green).
Throughout, numbers less than 10 should be written and not numeric
Thanks for pointing this out. The change was made throughout the text (highlighted in green).

Line 193 (and throughout), every organism has a phenotype. It is either wild-type or mutant. I think the authors mean mutant phenotype here
Change was made throughout the text (highlighted in green).
Line 206, please provide references
Thanks for pointing to this omission. References are now included in line 233.
Line 243, extra space after 16
Change was made (line 271)

Point by point response to reviewer 2

(All changes to address reviewer 2 's comments are highlighted in blue in the new version of the manuscript)

5% fructose impact on longevity and mobility healthspan has been in a 2020 paper that focused on mitochondrial outcomes (PubMed 33258406). Roles as a high fat model are novel to the work here and merit original publication; documentation is of higher rigor than existing literature, and extends phenotyping in important ways. However, the authors should cite this work, and briefly summarize findings. The authors might convert the 5% value to compare to the 10mg/ml concentration used in this study in comments.
We thank the reviewer for pointing us to this publication. We now refer to this study in the introduction (lines 119 to 121), and in the discussion (lines 705-726). Also, when we first mention fructose supplementation in the results section, we now include the fructose concentration as percent concentration, which is 1% in our study (line 365).
Likewise, PMID 27680107 reports on low dose fructose for lifespan extension; it is of interest to the field for He et al to cite this and to point these data out, with comment if possible.
We again thank the reviewer for pointing us to this publication. We now cite the findings of this work in the introduction (lines 119-123) and the discussion (lines 705-726).<br><br>We also briefly describe our thoughts here.<br>Seemingly distinct from our work, Zheng et al. (PMID 27680107) reported that supplementing 1% fructose extends *C. elegans* lifespan. However, there are experimental details that may explain the discrepancy.

Zheng et al supplemented 1% fructose to worm media plates seeded with *E. coli* OP50, while we used plates seeded with *E. coli* HT115. We made the choice of performing our whole study on animals fed HT115 to maintain the dietary composition consistent between the primary screen and the follow up studies. The relevance of *E. coli* to *C. elegans* metabolic phenotypes is well documented. Fat storage, healthspan, and lifespan depends on the bacteria composition (Brooks et al., 2009; Soukas et al., 2009; Stuhr and Curran, 2020). Further, recent large-scale *E. coli* mutant screens revealed microbial biochemical pathways influencing *C. elegans* fat accumulation (Neve et al., 2020) and longevity (Han et al., 2017). Based on these studies, we hypothesize that the discrepancies in the *C. elegans* lifespan phenotypes when fed the same dose of fructose may be due to *E. coli*-driven effects. This is a very interesting observation since little is known about the molecular underpinnings defining the outcomes of different diet-microbiome-host three-way interactions in the development of obesity. Therefore, our *C. elegans* HFrD model can serve as an informative platform to molecularly dissect such complex interactions in future studies.

The authors document that use of an rrf-3 mutant to potentiate RNAi does not change baseline fat phenotypes, an important control. Still, the targeted screen in this background might miss critical genes expressed in neurons and rrf-3 deficits could change the interactions with the genes identified. Minimally, the authors should discuss this caveat relative to outcome. It would be informative to test in wt background, and someday (not for this paper) retest the candidate set in a more directed neuronally-sensitized strain.

We appreciate the reviewer's comment on this matter since we had overlooked pointing to these caveats, which are now addressed in lines 189-198, and 650-653.

For further clarification, we used the RNAi-sensitized background *rrf-3* precisely because it has been shown to overall increase the penetrance of RNAi (Ceron et al., 2007; Hamilton et al., 2005; Simmer et al., 2003), including in neurons (Simmer et al., 2002). In particular, using the *rrf-3* background, (Lejeune et al., 2012) identified neuronal genes and pathways contributing to disease outcomes in a *C. elegans* model of Huntington's disease (Lejeune et al., 2012). Nevertheless, we agree with the reviewer that some targets may be RNAi-inaccessible even in this background, and most definitively that gene-gene and gene-environment interactions may be altered by mutation of *rrf-3*. As such, the choice of genetic background (*rrf-3*) may limit or skew the discovery of genes with background-specific phenotypic effects (e.g. only evident in metabolically sensitized backgrounds such as deficient insulin or serotonin signaling (Ashrafi et al., 2003; Samuelson et al., 2007)) or genes only penetrant in the wild type background. At the very least, altering the endogenous RNAi pathways impacts the *C. elegans* response to stress (Bharadwaj and Hall, 2017), which may affect *C. elegans* fat storage capacity under certain conditions.

On the other hand, the reviewer’s comment on testing our candidates in neuronally sensitized strains is a great suggestion in line with our plans for future studies. As evident by this study (Fig. 6), genes promoting DIO show tissue specific variations on fat allocation. Assuming that at least some of the hit genes will have cell-autonomous functions, the observation suggests that the obesity genes may have tissue specific functions in fat metabolism.

The senior author is well known for her contributions to the use of Oli red O for analysis of C. elegans fat stores, and her work highlighted that this reagent detects fat in intestine, hypodermis, germline and eggs. Thus, lack of germline/eggs, i.e. the sterile class of animals, might be anticipated to have low staining—that 9 genes for which kd enhances fat actually increase is therefore particularly striking, and suggests a possible change in fat tissue distribution when germline/eggs are lacking. The authors might note this mechanistic possibility when commenting on the obesity/infertility connection.
We thank the reviewer for this comment.<br><br>A soma-to-germline tradeoff has been shown to influence healthspan and lifespan in multicellular organisms (*i.e.*, disposable soma theory, summarized in review by (Maklakov and Immler, 2016). However, little is known about the molecular underpinnings of this trade-off. As suggested by the reviewer, future study of the sterile/obese phenotypic class may shed light onto a poorly understood but biologically fundamental question: how is the soma-to-germline tradeoff controlled and executed in multicellular organisms?<br><br>We now discuss the above notions in lines 228 to 231
That puf-8 stands out as the exception to the joint disruption of fertility and fat accumulation is interesting; a bit more comment on that issue could be warranted.
We appreciate the reviewer's comment on this. We have addressed this matter in lines 223-228.<br><br>In doing a more in depth literature search on *puf-8*, we found that it is actually important in regulating germline stem and progenitor cell development (summarized in a recent review by (Wang and Voronina, 2020). Further, *puf-8* deficiency has been linked to germline phenotypes including reduced germline and progeny size and a tumorous germline (Bachorik and Kimble, 2005; Subramaniam and Seydoux, 2003). Therefore, our "non-sterile" classification of *puf-8* may arise from our simplified binary "sterile versus fertile" classification, which must not imply lack of fertility disruption in animals showing oocytes and embryos. If confirmed with a null mutant, the quantitative (as opposed to qualitative) effect of *puf-8* on fertility can be used to more finely dissect the mechanisms defining soma to germline nutrient allocation in the future.
Lines 297, 298 that human and elegans homologs are regulated in the same direction is interesting and worth pointing out. Still, correlations do not exactly speak to "conserved evolutionary function"—it is more precise to say conserved regulation.
We appreciate the reviewer's comment as it shows our interpretation of the correlation analysis was not clearly described. We have now changed the text to clarify our interpretation of the conserved correlations between expression levels and the fat phenotype in humans, mice, and *C. elegans*.<br>Please find changes in lines 329-338 of the revised manuscript.

Page 13 section regarding Figure 3C is a bit unclear. The legend refers to published GWAS studies, the narrative in the text refers to the not-yet-cataloged MRTSIM loci but the analysis relies on what are in the published GWAS —can the authors clarify the main point of this panel in the text and legend? I take it that 15/16 of genes identified have published GWAS data that links them to metabolic measures; what is the main point of the pleiotropic effects? This might be more clearly articulated.
We thank the reviewer for pointing out the lack of clarity on the message we intended to convey with Fig. 3C.<br><br>The goal of this figure panel is to summarize current data that may indicate which of the hit genes are more likely to have metabolic-specific or metabolic-related functions, and which ones are more likely to be pleiotropic genes. For instance, many genes that just generally make animals sick may promote leanness.<br>To illustrate our point. *ADAMTS9* and *ZFHX3* have been reported as hits in a similar number of GWAS studies. Nevertheless, *ADAMTS9* variants are mostly associated with metabolic traits whereas *ZFHX3* variants are mostly associated with non-metabolic traits. Based on the rationale presented above, we'd propose that *ADAMTS9* is more likely to play metabolic-specific or metabolic-related functions than *ZFHX3*.<br><br>The notions described above are now included in the manuscript in lines 279-281, 308-314, 340-345.
Line 364, discussion around line 373, again 533—reference to the long lived daf-2 mutant having elevated fat stores. If high fat tends to be associated with short life, the daf-2 mutant shows the opposite. Some discussion of this paradox might be informative; at least the paradox might be recognized.
We appreciate the reviewer's comments on this paradox, which remains unsolved. We speculate that the generalized enhancement of cellular homeostatic mechanisms observed in *daf-2* animals, ranging from improved mitochondrial and ER health to increased autophagy and proteostasis, would compensate for the detrimental effects of excessive fat storage. Alternatively, since different lipids have different effects on cellular and organismal health (e.g. saturated versus unsaturated fats), the classes of fats accumulating in *daf-2* animals could be different from those accumulating in high-fructose fed animals.<br><br>Nevertheless, as part of the general changes introduced in the new version of the manuscript, we had removed the references to *daf-2* in those lines, and replaced it with a reference to the mTORC2-component *rict-1* (Line 409-410). Loss of *rict-1* function promotes excessive fat storage across tissues and reduces *C. elegans* lifespan (Soukas et al., 2009; Yen et al., 2010). Further, similar to the differences between the observations reported in Zheng *et al* and our study on the effect of fructose on *C. elegans* lifespan, Soukas *et al* showed that changing the *E coli* strain from OP50 to HT115 reverts the effect of *rict-1* mutation on *C. elegans*'s fat and lifespan phenotypes (Soukas et al., 2009; Yen et al., 2010).<br><br>On the other hand, we refer to *daf-2* to address comment # 2 of Reviewer 3 (lines 419-427). *daf-2* mutants show increased body fat content with a concomitant extension in lifespan, which demonstrates that excessive fat storage can be uncoupled from the detrimental effects of fat on health and lifespan. Thus, we use the *daf-2* example to argue in favor of testing the

effect of inactivating our “lean genes“on *C. elegans* healthspan and lifespan.
Line 407 wording is awkward. Better: We tested the 293 C. elegans orthologs of the human obesity GWAS candidate genes for DIO causality by screening…..
We changed the text as suggested by the reviewer (lines 461 to 462).

Point by point response to reviewer 3

(All changes to address reviewer 3 ’s comments are highlighted in yellow in the new version of the manuscript)

1. The identification of the 293 genes from obesity GWAS is quite brief in the paper. It would be helpful to the reader if the authors would provide a bit more detail into how they got to the 293 genes.
We very much appreciate your suggestion. We have now extended the description of how we arrived at the final list of 293 genes to be screened (lines 148-169, 184-189). Readers will also find a graphical abstract in Fig. 1A.<br><br>Briefly, we derived our human gene list from 3 papers. The first, from our group, identified genetic variants associated with gene expression in adipose tissue using eQTL analysis (Civelek et al., 2017). We then found variants associated with obesity and fat distribution in GWAS. This resulted in 211 high quality gene candidates. Second, we used variants found in an obesity GWAS of Japanese and multi-ethnic ancestry (Akiyama et al., 2017). They found 120 genes nearby novel variants. Finally, a GWAS of ectopic fat deposition identified 11 novel genes (Chu et al., 2017). In total, we started with 340 human genes associated with obesity. 207 of the human genes had orthologs in *C. elegans* and 293 *C. elegans* orthologs mapped to the 207 human genes. We have better explained this description in the results section.
2. What is the logic of testing all the co-morbidities and healthspan parameters in the HFrd system? I had a bit of trouble following the logic in the paper for this.
Thank you for bringing up this point, which was not clearly explained in the first version of the manuscript.<br><br>Obesity is defined by the World Health Organization as abnormal or excessive fat accumulation that presents a higher risk of debilitating co-morbidities and death (Ahima, 2009; Salvestrini et al., 2019; Tam et al., 2020). In *C. elegans* and mammals, excessive fat accumulation is not always associated with deleterious effects on health. For instance, inactivation of *C. elegans* insulin receptor (IR) *daf-2* leads to excessive fat accumulation (O’Rourke et al., 2009). However, these mutant animals are long lived and resistant to a wide-

range of age-related diseases (Halaschek-Wiener et al., 2005; Kimura et al., 1997). Consistently, GH receptor knockout (GHRKO) mice have increased fat mass and decreased lean mass; however, they show improved insulin sensitivity and are long lived (Fang et al., 2020). Therefore, since obesity can be genetically uncoupled from its detrimental effects on health and lifespan, an instructive invertebrate model of obesity must satisfy the requirement of having poor health outcomes and shorter lifespan, and a gold-standard suppressor gene would be one that suppresses obesity and its associated detrimental effects on health and lifespan.<br><br>For these reasons we tested whether the genes suppressing DIO in *C. elegans* were also able to ameliorate health outcomes and extend the lifespan of animals feeding a high-fructose diet.<br><br>We included the notions described above in lines 419-427, 589-590, and 747-755 of the revised manuscript
3. As many of the people reading this paper would be interested in the human gene name, it would be helpful to see those gene names integrated into the text earlier on in the manuscript.
Thank you for this suggestion as it will make our manuscript more accessible to a broader readership. We have incorporated the human gene names into the text when they are first mentioned (line 219-221, 236-237, 251-254, 477, 505), as well as in Figures 3A, 3B.
4. Could you show the human locus plots for the key genes you validate in your study?
Thank you for the suggestion. We have now included locus zoom plots for each gene that we discuss in both WHRadjBMI GWAS and BMI GWAS. We describe the results in lines 345-348 and in methods section lines 908-910. The plots can be found in the new Suppl. LocusPlots S1 and S2.
5. Certainly there are limitations of using c. elegans in studying such a complex trait as obesity. It would be helpful to have a formal paragraph on the limitations of the model.
We very much appreciate this comment. We have included a paragraph in the discussion section describing the limitations of an invertebrate model system as well as of the methodology (lines 637-655).

References

Ahima, R.S. (2009). Connecting obesity, aging and diabetes. Nat. Med. *15*, 996–997.

Akiyama, M., Okada, Y., Kanai, M., Takahashi, A., Momozawa, Y., Ikeda, M., Iwata, N., Ikegawa, S., Hirata, M., Matsuda, K., et al. (2017). Genome-wide association study identifies 112 new loci for body mass index in the Japanese population. Nat. Genet. *49*, 1458–1467.

Ashrafi, K., Chang, F.Y., Watts, J.L., Fraser, A.G., Kamath, R.S., Ahringer, J., and Ruvkun, G. (2003). Genome-wide RNAi analysis of Caenorhabditis elegans fat regulatory genes. Nature *421*, 268–272.

Bachorik, J.L., and Kimble, J. (2005). Redundant control of the Caenorhabditis elegans sperm/oocyte switch by PUF-8 and FBF-1, two distinct PUF RNA-binding proteins. Proc Natl Acad Sci USA *102*, 10893–10897.

Bharadwaj, P.S., and Hall, S.E. (2017). Endogenous RNAi Pathways Are Required in Neurons for Dauer Formation in Caenorhabditis elegans. Genetics *205*, 1503–1516.

Brooks, K.K., Liang, B., and Watts, J.L. (2009). The influence of bacterial diet on fat storage in C. elegans. PLoS ONE *4*, e7545.

Ceron, J., Rual, J.-F., Chandra, A., Dupuy, D., Vidal, M., and van den Heuvel, S. (2007). Large-scale RNAi screens identify novel genes that interact with the C. elegans retinoblastoma pathway as well as splicing-related components with synMuv B activity. BMC Dev. Biol. *7*, 30.

Chu, A.Y., Deng, X., Fisher, V.A., Drong, A., Zhang, Y., Feitosa, M.F., Liu, C.-T., Weeks, O., Choh, A.C., Duan, Q., et al. (2017). Multiethnic genome-wide meta-analysis of ectopic fat depots identifies loci associated with adipocyte development and differentiation. Nat. Genet. *49*, 125–130.

Civelek, M., Wu, Y., Pan, C., Raulerson, C.K., Ko, A., He, A., Tilford, C., Saleem, N.K., Stančáková, A., Scott, L.J., et al. (2017). Genetic Regulation of Adipose Gene Expression and Cardio-Metabolic Traits. Am. J. Hum. Genet. *100*, 428–443.

Fang, Y., McFadden, S., Darcy, J., Hascup, E.R., Hascup, K.N., and Bartke, A. (2020). Lifespan of long-lived growth hormone receptor knockout mice was not normalized by housing at 30°C since weaning. Aging Cell *19*, e13123.

Halaschek-Wiener, J., Khattra, J.S., McKay, S., Pouzyrev, A., Stott, J.M., Yang, G.S., Holt, R.A., Jones, S.J.M., Marra, M.A., Brooks-Wilson, A.R., et al. (2005). Analysis of long-lived C. elegans daf-2 mutants using serial analysis of gene expression. Genome Res. *15*, 603–615.

Hamilton, B., Dong, Y., Shindo, M., Liu, W., Odell, I., Ruvkun, G., and Lee, S.S. (2005). A systematic RNAi screen for longevity genes in C. elegans. Genes Dev. *19*, 1544–1555.

Han, B., Sivaramakrishnan, P., Lin, C.-C.J., Neve, I.A.A., He, J., Tay, L.W.R., Sowa, J.N., Sizovs, A., Du, G., Wang, J., et al. (2017). Microbial genetic composition tunes host longevity. Cell *169*, 1249-1262.e13.

Kimura, K.D., Tissenbaum, H.A., Liu, Y., and Ruvkun, G. (1997). daf-2, an insulin receptor-like gene that regulates longevity and diapause in Caenorhabditis elegans. Science *277*, 942–946.

Lejeune, F.-X., Mesrob, L., Parmentier, F., Bicep, C., Vazquez-Manrique, R.P., Parker, J.A., Vert, J.-P., Tourette, C., and Neri, C. (2012). Large-scale functional RNAi screen in C. elegans identifies genes that regulate the dysfunction of mutant polyglutamine neurons. BMC Genomics *13*, 91.

Maklakov, A.A., and Immler, S. (2016). The expensive germline and the evolution of ageing. Curr. Biol. *26*, R577–R586.

Neve, I.A.A., Sowa, J.N., Lin, C.-C.J., Sivaramakrishnan, P., Herman, C., Ye, Y., Han, L., and Wang, M.C. (2020). Escherichia coli Metabolite Profiling Leads to the Development of an RNA Interference Strain for Caenorhabditis elegans. G3 (Bethesda) *10*, 189–198.

O'Rourke, E.J., Soukas, A.A., Carr, C.E., and Ruvkun, G. (2009). C. elegans major fats are stored in vesicles distinct from lysosome-related organelles. Cell Metab. *10*, 430–435.

Salvestrini, V., Sell, C., and Lorenzini, A. (2019). Obesity may accelerate the aging process. Front Endocrinol (Lausanne) *10*, 266.

Samuelson, A.V., Carr, C.E., and Ruvkun, G. (2007). Gene activities that mediate increased life span of C. elegans insulin-like signaling mutants. Genes Dev. *21*, 2976–2994.

Simmer, F., Tijsterman, M., Parrish, S., Koushika, S.P., Nonet, M.L., Fire, A., Ahringer, J., and Plasterk, R.H.A. (2002). Loss of the putative RNA-directed RNA polymerase RRF-3 makes C. elegans hypersensitive to RNAi. Curr. Biol. *12*, 1317–1319.

Simmer, F., Moorman, C., van der Linden, A.M., Kuijk, E., van den Berghe, P.V.E., Kamath, R.S., Fraser, A.G., Ahringer, J., and Plasterk, R.H.A. (2003). Genome-wide RNAi of C. elegans using the hypersensitive rrf-3 strain reveals novel gene functions. PLoS Biol. *1*, E12.

Soukas, A.A., Kane, E.A., Carr, C.E., Melo, J.A., and Ruvkun, G. (2009). Rictor/TORC2 regulates fat metabolism, feeding, growth, and life span in Caenorhabditis elegans. Genes Dev. *23*, 496–511.

Stuhr, N.L., and Curran, S.P. (2020). Bacterial diets differentially alter lifespan and healthspan trajectories in C. elegans. Commun. Biol. *3*, 653.

Subramaniam, K., and Seydoux, G. (2003). Dedifferentiation of primary spermatocytes into germ cell tumors in C. elegans lacking the pumilio-like protein PUF-8. Curr. Biol. *13*, 134–139.

Tam, B.T., Morais, J.A., and Santosa, S. (2020). Obesity and ageing: Two sides of the same coin. Obes. Rev. *21*, e12991.

Wang, X., and Voronina, E. (2020). Diverse Roles of PUF Proteins in Germline Stem and Progenitor Cell Development in C. elegans. Front. Cell Dev. Biol. *8*, 29.

Yen, K., Le, T.T., Bansal, A., Narasimhan, S.D., Cheng, J.-X., and Tissenbaum, H.A. (2010). A comparative study of fat storage quantitation in nematode Caenorhabditis elegans using label and label-free methods. PLoS ONE *5*.
